# The EEG analysis and identification of Alzheimer's disease: a review

**DOI:** 10.3389/fnagi.2025.1686628

**Published:** 2025-12-09

**Authors:** Jinying Bi, Fei Wang, Fangzhou Hu, Shuai Han, Yuting Wang, Zhijian Fu, Xin Zhang

**Affiliations:** 1 Faculty of Robot Science and Engineering, Northeastern University, Shenyang, China; 2 Department of Neurosurgery, Shengjing Hospital of China Medical University, Shenyang, China; 3 Product Innovation Center, Shenyang Ruijin Medical Technology Company Limited, Shenyang, China; 4 Office of the President, Shenyang First People's Hospital, Shenyang, China; 5 Department of Rehabilitation Medicine, Shenyang First People's Hospital, Shenyang, China

**Keywords:** Alzheimer's disease (AD), neurodegenerative disorder, electroencephalogram (EEG), AD identification, biomarker

## Abstract

Alzheimer's disease (AD), a neurodegenerative disorder, significantly impacts patients, families, and society. Therefore, efficient AD diagnosis and disease analysis are crucial. Electroencephalogram (EEG) directly reflects brain activity, making EEG-based AD identification a current research hotspot. This review utilized digital libraries (Google Scholar and PubMed) to categorize the literature into two sets based on different periods, ultimately analyzing the application of EEG in AD research through 141 articles after screening. Critical topics addressed include subject types, experimental design, electrode selection, artifact processing, rhythm division, feature extraction, recognition methods, etc. Additionally, the review discusses major conclusions, emphasizing research priorities and consistent findings. The study also briefly mentions other biomarkers and predicts future trends of EEG as a biomarker. This work provides valuable references for researchers and clinicians exploring the relationship between EEG and AD.

**Systematic review registration:**

https://www.crd.york.ac.uk/PROSPERO/.

## 1 Introduction

As the aging population increases, the impact of degenerative diseases on public health becomes more severe. The world health organization estimates that over 55 million people worldwide suffer from severe cognitive impairment, known as dementia (Better, 2023). This common neurodegenerative disorder includes Subjective Cognitive Decline (SCD), Mild Cognitive Impairment (MCI), Dementia with Lewy Bodies (DLB), Frontotemporal Dementia (FTD), Vascular Cognitive Impairment (VCI), and Alzheimer's Disease (AD) (Rossor et al., 2016). Once diagnosed, experience long-term irreversible cognitive decline, with AD accounting for 60–70% (Better, 2023) of dementia cases. The symptoms mainly manifest as memory decline, cognitive impairment, and behavioral dysfunction, significantly affecting self-care abilities and impacting families, society, and economic development.

The diagnosis of AD has become a significant public health and social issue. Factors (James and Bennett, 2019) such as age, genetics (Scheltens et al., 2021), head injuries, neuroinflammation, and hormonal imbalances may contribute to AD onset. However, though it is commonly hypothesized that AD is related to amyloid-beta (*Aβ*) accumulation in cerebrospinal fluid (CSF) and tau protein abnormalities (Hampel et al., 2021; Sehar et al., 2022), the exact mechanisms remain unclear, complicating diagnosis. While the Mini-Mental State Examination (MMSE) (Arevalo-Rodriguez et al., 2021) is a preliminary tool for monitoring cognitive impairment, its limitations necessitate advanced imaging techniques like Magnetic resonance imaging (MRI) (Chandra et al., 2019) and positron emission tomography (PET) (Chételat et al., 2020; Zhou et al., 2021) in-depth analysis. Although PET is more accurate, its high cost deters many patients (Contador et al., 2023), making lower-resolution MRI the preferred choice. However, errors in medical imaging diagnoses are increasing with more complex cases. Invasive methods, such as CSF analysis and brain tissue biopsy, are rarely used. (Khan et al., 2020). Sample diversity complicates AD diagnosis. Current treatments can only delay progression to severe stages (Zvěřová, 2019), highlighting the importance of early identification to improve patient quality of life.

Brain signals reflecting neural activity show promise for early AD detection, potentially allowing for prompt interventions. Researchers are increasingly focusing on neural signals, such as electroencephalogram (EEG) and functional Near-Infrared Spectroscopy (fNIRS). EEG-based studies are particularly prominent, but differing methodologies have led to variability in conclusions, preventing widespread clinical application of EEG biomarkers for AD. The design of paradigms for EEG collection, signal preprocessing, rhythmic analysis, feature selection, and model analysis has become the main steps in analyzing AD biomarkers. Resting-state EEG signals have been shown to provide valuable insights into cognitive responses of AD patients to interventions like transcranial direct current stimulation (tDCS) (Andrade et al., 2023). Clinically, some researchers (Babiloni et al., 2024; Meghdadi et al., 2024; Amato et al., 2024; Ge et al., 2025) have conducted follow-up analyses of EEG from patients, but the generalizability of these conclusions is limited. Therefore, the non-invasive EEG-based AD biomarkers still requires further exploration.

**Figure 1:**
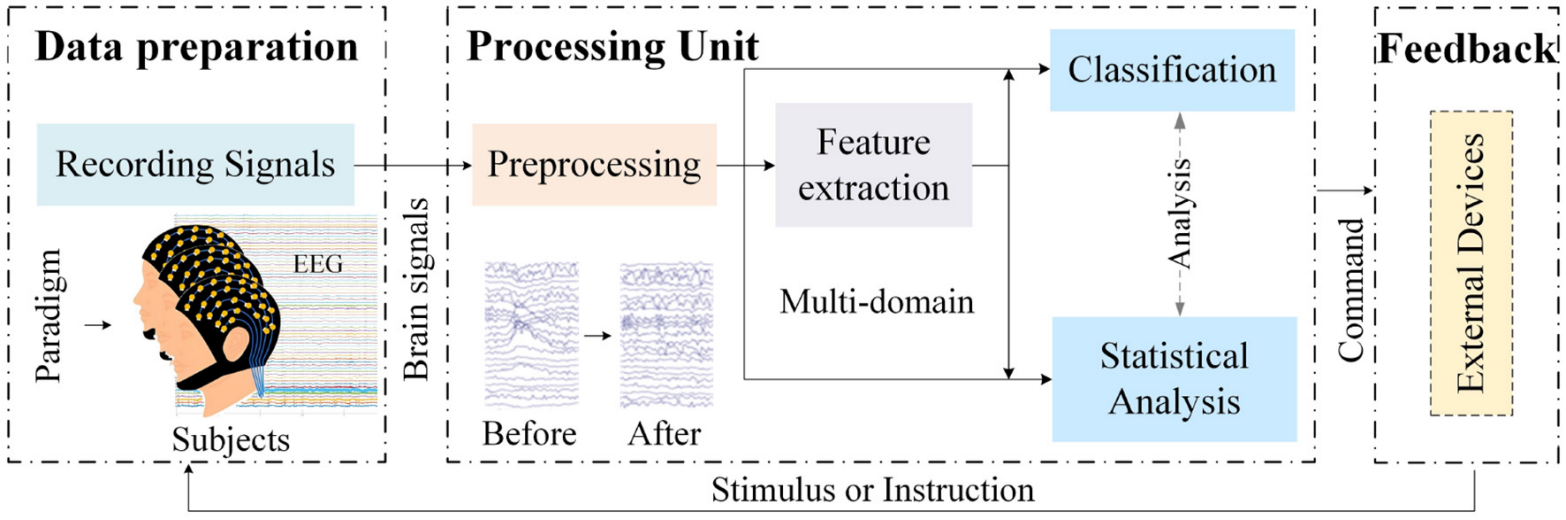
The basic components of a BCI system.

**Figure 2:**
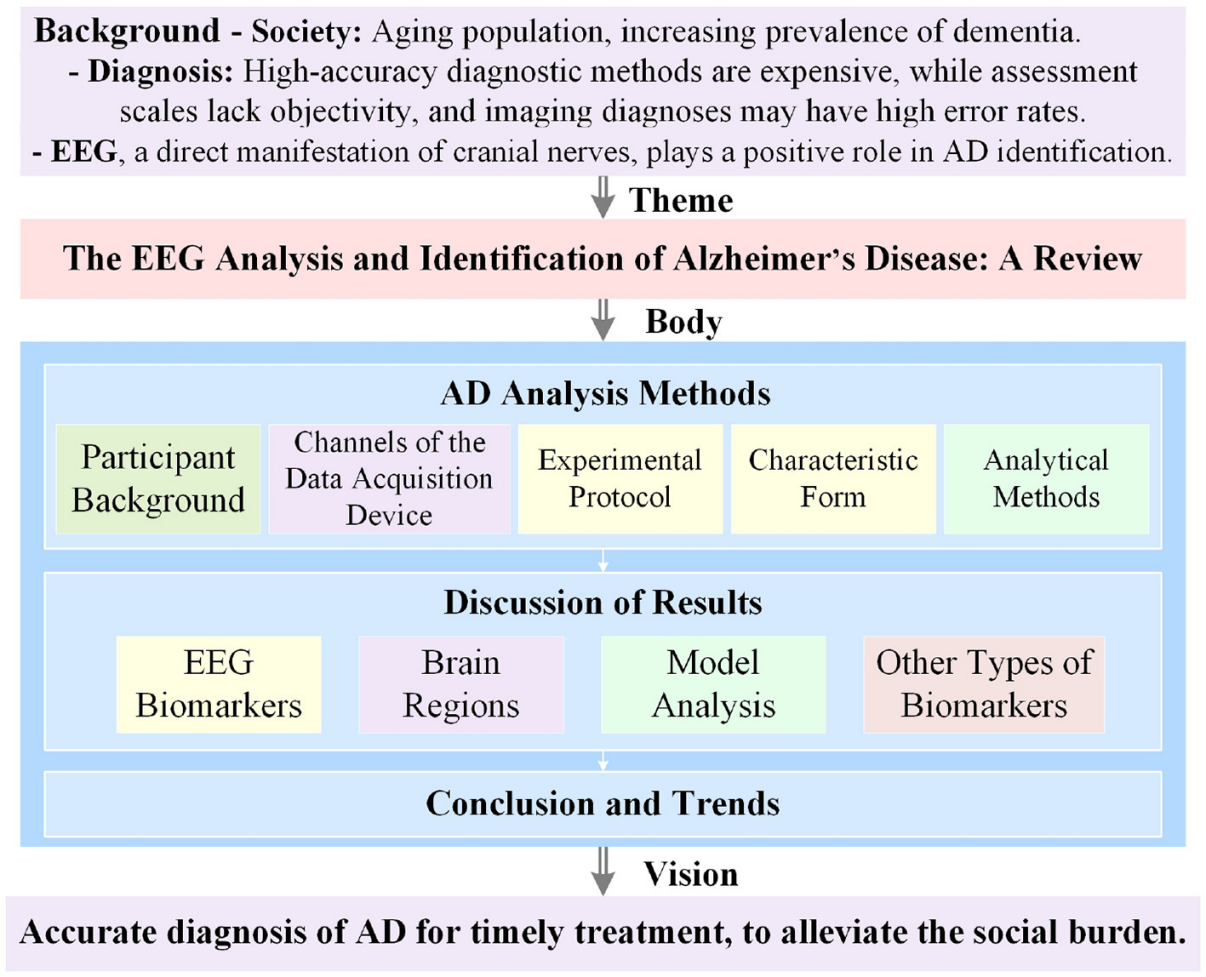
Basic overview of the review. “Body” provides details of the first two parts of the BCI system for AD.

The identification route of brain-computer interfaces (BCI) based on AD is nearly identical to that of conventional BCI routes. Typical components of a BCI system are shown in Figure 1. The paradigm type of EEG-based AD recognition systems is designed with various evoked designs and falls under the category of passive BCIs. However, this AD recognition system focuses more on discrimination, leaning toward a “read-only” BCI pipeline. This indicates that, in the traditional BCI analysis process, it places greater emphasis on data preparation and processing units, while feedback components typically consist of some means for controlling external devices and therapeutic stimuli, which are often absent in this process. Researchers are increasingly focused on identifying gold standard EEG biomarkers for AD recognition. This review systematically examines recent research on EEG biomarkers for AD, summarizing and discussing relevant articles and outlining future development trends. This overview is shown in Figure 2.

The manuscript is organized as follows: Section 1 is the Introduction; Section 2 presents the search strategy for the review articles; Section 3 describes the AD analysis methods; The discussion of results will be covered in Section 4; Finally, Section 5 is the conclusion.

## 2 Search strategy

**Figure 3:**
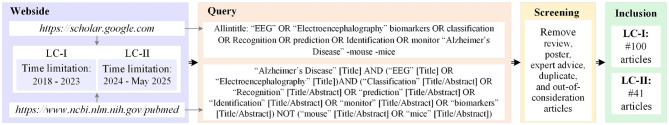
Preparation steps for literature screening: identification (Website, Query), screening, and inclusion. The process reduced LC-I from an initial total of 108 + 92 = 200 articles to 100 articles, and LC-II from 40 + 41 = 81 articles to 41 articles related to EEG and Alzheimer's disease.

The review focuses on EEG-based identification of AD by searching for relevant keywords on *Google Scholar* and *PubMed* to obtain pertinent articles. The exploration of EEG analysis and identification of AD gradually increased in 2018 and saw a significant surge in 2024. To clearly highlight the current research trends and key points, this review categorizes the collected articles into two literature sets based on their publication dates. The literature is organized into two collections: Literature Collection I (LC-I) includes articles from 2018 to 2023, while Literature Collection II (LC-II) contains articles from 2024 to May 2025. The literature search and inclusion process is illustrated in Figure 3.

This review incorporates articles identified through keyword searches in article databases (**Google Scholar**: allintitle: “EEG” OR “Electroencephalography” biomarkers OR classification OR Recognition OR prediction OR Identification OR monitor “Alzheimer's disease” -mouse -mice; **PubMed**: “Alzheimer's disease”[Title] AND (“EEG”[Title] OR “Electroencephalography”[Title]) AND (“classification”[Title/Abstract] OR “‘Recognition”[Title/Abstract] OR “prediction”[Title/Abstract] OR “Identification”[Title/Abstract] OR “monitor”[Title/Abstract] OR “biomarkers”[Title/Abstract]) NOT (“mouse”[Title/Abstract] OR “mice”[Title/Abstract]))).

To ensure a more uniform search scope for the literature collection, this review excludes the following types of articles: duplicate articles from the two digital libraries, review articles, expert recommendations, books, posters, studies exploring cerebrospinal fluid treatments, and articles related to medication and drug metabolism. The specific literature search and inclusion details for LC-I and LC-II are as follows:

1. LC-I (Google Scholar): 108 articles were found - 6 reviews - 2 posters - 1 book - duplicate articles - articles that were non-eligible = 60 articles.
2. LC-I (PubMed): 92 articles were found - 14 reviews - 1 expert group recommends articles - duplicate articles - articles that were non-eligible = 40 articles.

Therefore, LC-I consists of 100 articles (60 + 40), with authors from various countries (China, the United States, Korea, Japan, India, and others).

1. LC-II (Google Scholar): 40 articles were found - articles that were non-eligible = 28 articles.
2. LC-II (PubMed): 41 articles were found - 5 reviews - 16 duplicate articles - 1 preprinted article - articles that were non-eligible = 13 articles.

Hence, this review includes 41 articles (28 + 13) in LC-II.

In summary, this review mainly examines 141 articles to provide an overview and brief discussion.

## 3 Alzheimer's disease analysis methods

Based on the exploration of dementia research in LC-I and LC-II, this section summarizes the analysis into five parts. Provide a **reference-style brief summary** for subpoints.

### 3.1 Participants' background

Currently, the relationship between the occurrence of AD and patient background is not clearly defined. This section will outline the age, education level, gender, and total number of participants mentioned in the literature.

#### 3.1.1 Aging

- *Literature Collection I:*

The dataset indicates that the average participant age is over 55 years, focusing on the early middle-aged and elderly population in dementia research. One dataset includes some healthy adolescents for comparison purposes (Meghdadi et al., 2021b).

- *Literature Collection II:*

Compared to LC-I, LC-II does not include adolescent participants in the dataset for EEG studies on AD. All participants belong to the middle-aged and older adult populations.

- ***Reference-style brief summary***

Similarities: Both literature sets focus on middle-aged and elderly populations. Differences: The LC-I includes comparative studies with adolescents. Connections: Adolescents have a low probability of developing AD. Using them as a control group may overlook the natural progression of neurodegenerative aging. Comparing people in the same age range provides stronger evidence. The risk of developing AD may increase with age. Therefore, research on middle-aged and elderly populations is more beneficial for exploring AD.

#### 3.1.2 Education level

- *Literature Collection I:*

The educational level of participants influences the likelihood of developing AD (Babiloni et al., 2021). In LC-I, fewer than 25 articles considered education, typically measured in years. Some categorize education into levels (Ruiz-Gómez et al., 2018b; Tzimourta et al., 2019), such as primary, secondary, and higher. However, educational level has received limited attention in specific AD analyses.

- *Literature Collection II:*

In LC-II, the educational background of participants has not been a primary focus. Most articles do not even mention the years of education of the subjects within the dataset.

- ***Reference-style brief summary***

Similarities: Some studies in both literature sets provide information on the years of education of the participants. Differences: In the LC-I, there are classifications for measuring educational levels, whereas the LC-II set does not emphasize years of education as much. Connections: Research (Seyedsalehi et al., 2023) has shown that lower education levels are linked to a higher risk of developing the disease. Educational level serves as a “hidden” label for research groups. However, the current studies do not focus on this aspect. Therefore, while it may not be emphasized in the collection of patient information, if in-depth analysis in this domain is desired, it could warrant separate statistical examination.

#### 3.1.3 Gender

- *Literature Collection I:*

**Figure 4:**
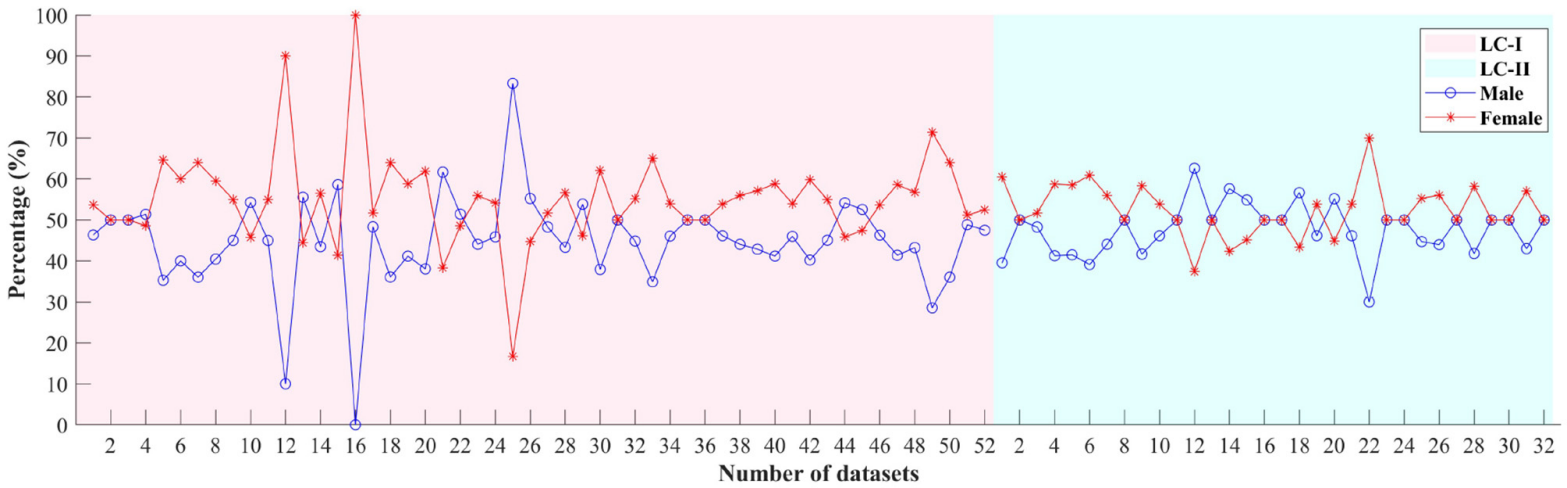
Gender ratio diagram for each dataset in LC-I and LC-II.

Some articles do not specify participant numbers by gender. This section summarizes the available data. Figure 4 illustrates the percentage of gender across datasets in LC-I and LC-II. The x-axis represents the dataset number, while the y-axis shows the percentage of participants in each dataset.

The ratio line chart for the first 52 datasets (pink background) in Figure 4 indicates that female participants generally outnumber males, though some datasets show a balanced gender ratio. On average, males account for 43.95% and females for 56.07%. However, nearly half of the datasets lack clear gender descriptions, and while some mention gender considerations, specific values are often missing.

- *Literature Collection II:*

The line chart for the last 20 datasets (blue background) in Figure 4 shows the available gender proportion in LC-II. The overall average percentage is 47.63% for males and 52.37% for females. Together with LC-I, the data suggests that researchers prefer datasets with a balanced male-to-female ratio.

- ***Reference-style brief summary***

Similarities: In both studies, the overall proportion of female participants is higher than that of male participants, suggesting a potentially higher incidence of the disease in women. Differences: The selection criteria in the LC-II are more oriented toward achieving a balanced gender ratio. Connections: Although the gender distribution indicates that female subjects outnumber males, it remains unclear whether the gender of the study participants is randomized. Therefore, to minimize gender-related interference in EEG data, it is advisable to maintain a gender ratio balance during data collection.

#### 3.1.4 Participants count and group categories

- *Literature Collection I:*

In AD research, most studies incorporate a control group, while some focus solely on individuals with AD (Seo et al., 2020; Kayasandik et al., 2022; Andrade et al., 2023). Additionally, some publications analyze multiple AD-related datasets within a single study. In LC-I, most studies use healthy participants as controls for AD identification. However, a few articles explore the relationship between cognitive disorders and AD. This part will analyze participant numbers and categories based on all datasets with specified participant counts in LC-I.

**Figure 5:**
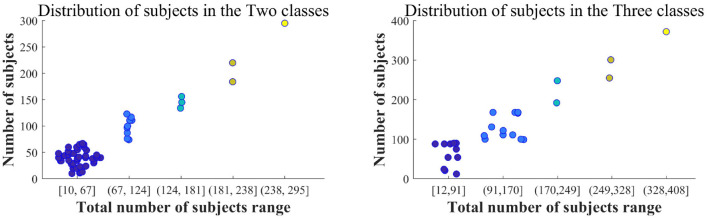
Distribution of population in two and three types of datasets in LC-I.

**Two classes:** The left of Figure 5 illustrates the gradient distribution for the two types of datasets, which include 10 to 295 participants, averaging around 64 per category. Most datasets consist of combinations of AD and healthy controls (HC), with a smaller number combining AD and other cognitive impairments.

**Three classes:** The dataset for the three categories range from 12 to 890 participants, averaging about 163 subjects per category. The total of 890 includes four dataset types, focusing on three. Excluding the fourth type, the range adjusts to 12–408, with an average of 147 subjects per category. The right of Figure 5 visually represents this dataset across five segments, detailing the frequency of the three subject types. Most datasets consist of combinations of AD, MCI, and HC, with some including (FTD, DLB, or other dementias) vs. AD vs. HC.

From the data in Figure 5, the total number of participants in the two types and three types of subjects is primarily below 67 and 170, respectively.

**Four classes:** Few studies involve four types of subjects, with nine articles providing descriptions. Participant counts range from 29 to 890, averaging about 196 per category. The counts in ascending order are 29, 38, 45, 58, 99, 123, 169, 318, and 890. These subjects primarily consist of combinations of AD, MCI, HC, and other forms of dementia.

**Five classes:** Datasets with five subjects' types (HC1 vs. HC2 vs. HC3 vs. MCI vs. AD) (Meghdadi et al., 2021b) are present in LC-I, where healthy participants are divided into three categories and combined with MCI and AD, totaling 325 participants [HC1, HC2, and HC3 are derived from the researchers' classification of cognitively HC (aged 18-90) into three stages based on age. This classification aims to better reflect the impact of different age groups on cognitive health].

**More classes:** Similarly, there are datasets with more categories. A total of 96 participants were collected (HC, mild AD, AD, DLB, Parkinson's disease, and memory problems with unclear diagnosis) (Meghdadi et al., 2020). The proportions for each type are 67 : 16 : 4 : 1 : 1 : 7. However, participant numbers vary significantly among these categories.

In LC-I, datasets with clearly stated participant numbers are mentioned 98 times. Among these, two types of subjects are referenced 57 times, accounting for 58.16%. Datasets with three types are mentioned 30 times, making up 30.61%. The remainder involves datasets with more than three categories. Overall, AD analysis currently focuses on two groups, particularly AD and HC datasets, which are the most mentioned.

- *Literature Collection II:*

In 40 references, 43 datasets are described, categorized into four cases based on the number of subject groups involved.

**One class:** Similar to LC-I, there is a small proportion of datasets focusing on specific populations. Here, only two datasets pertains to a long-term therapeutic progression study on AD and MCI, including 56 and 107 participants (Youn et al., 2024; Ge et al., 2025).

**Two classes:** There are 23 binary category datasets, primarily involving patients and control groups (HC vs. AD; AD vs. non-AD; MCI vs. control; SCD vs. AD; MCI vs. HC). Participant numbers ranges from 23 to 182, in ascending order: 23, 24, 27, 30, 32, 33, 40, 48, 55, 58, 65, 82, 92, 94, 109, 120, 123, 125, 143, and 182 (non-AD is considered to include all subjects with an MMSE score greater than 26).

**Three classes:** The exploration of three subject categories encompasses seventeen datasets, combining two types of dementia and a control group (HC vs. AD1 vs. AD2; HC vs. FTD vs. AD; HC vs. MCI vs. AD). Participant numbers range from 57 to 231, increasing as follows: 57, 86, 88, 119, 144, 168, and 231. Notably, the EEG dataset with 88 participants has been utilized in 11 instances, indicating its broader application potential. Moreover, two studies selected two categories from this dataset for secondary analysis (Goerttler et al., 2024; Sreedhar et al., 2025) (AD1 and AD2 are the researchers more detailed distinctions of AD: mild AD and moderate AD).

**Figure 6:**
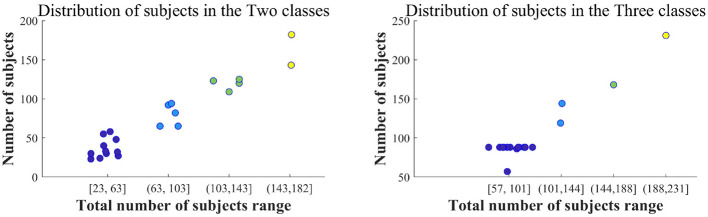
Distribution of population in two and three types of datasets in LC-II.

Figure 6 shows the gradient chart for the number of subjects in the Two class and Three class groups. It can be observed that the dataset for the two-class group, encompassing a total of 23 to 63 individuals, has been extensively studied. In the three-class study, the range of total subjects from 57 to 101 is given special attention. This data range is similar to that illustrated in LC-I. Therefore, in future research, the consideration of the number of subjects can be informed by this range.

**Four classes:** In LC-II, there is one dataset (SCD vs. NaMCI vs. aMCI vs. AD) (Kim S.-K. et al., 2024) of four groups, totaling 58 participants (NaMCI and aMCI are detailed subtypes of MCI: non-amnesic MCI and amnesic MCI).

- ***Reference-style brief summary***

Similarities: In both literature sets, the study groups predominantly focus on Categories two, three, and four. Differences: In two classes of LC-I and LC-II, the former tends to combine AD with control groups, while the latter features a greater diversity in participant labeling across these categories. Additionally, LC-I includes research with combinations exceeding four categories, whereas LC-II examines a maximum of four categories (different types of dementia). Connections: In EEG data analysis of AD, one category typically involves follow-up analyses of AD progression. However, in clinical practice, follow-up requires substantial participant compliance. As research advances, Categories II and III are increasingly prioritized by researchers. Two-group categorical data are primarily utilized to analyze and identify potential biomarkers between HC and patients. In contrast, three or more groups generally focus on differentiation between HC and various dementia populations. While having more subjects enhances analysis, maintaining sample balance remains crucial.

Background information of participants, considered as demographic variables, is well-documented in the literature. Age and gender are fixed characteristics of an individual's development, and neural changes can occur in relation to these factors. Environmental influences, such as education level, serve as important indicators of participants' cognitive activity. These demographic variables undoubtedly affect the neural activity of participants. Therefore, it is essential to maintain balance among these variables across categories during participant selection and assessment to eliminate potential confounding effects.

### 3.2 Channels of the data acquisition device

- *Literature Collection I:*

**Figure 7:**
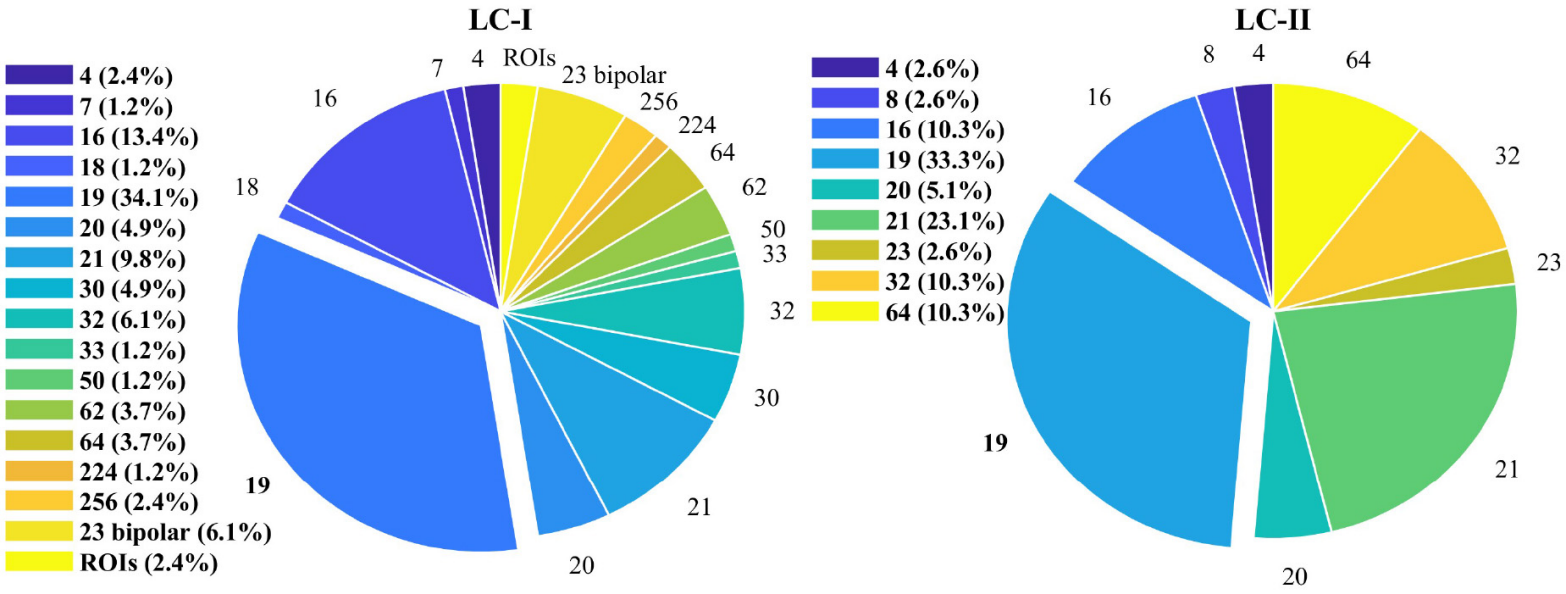
Proportion of articles in different channels numbers in LC-I and LC-II (the legend displays the percentage of articles corresponding to the number of applications for each channel combination).

In LC-I, 82 articles specifically analyze channel configurations, with counts of 4, 7, 16, 18, 19, 20, 21, 30, 32, 33, 50, 62, 64, 224, 256, and 23 bipolar electrodes, along with some regions of interest (ROIs). Notably, the 19-channel and 21-channel setups are similar. The 21 channels include the 19 plus 2 reference electrodes (Al-Nuaimi et al., 2021). The 62-channel comprises 60 EEG and 2 EOG channels (Ding et al., 2022). The 18-channel literature refers to 16 and 2 reference electrodes (Yu et al., 2019). The distribution of articles by channel count is shown in the pie chart ratio on the left in Figure 7.

Over one-third of the articles utilized the 19-channel dataset, followed by the 16-channel and 21-channel configurations. The 23 bipolar electrode channels were also commonly used. In the two articles (Seo et al., 2020; Dude et al., 2023) involving 4 channels, the specific channels of interest differ: one focuses on AF7, AF8, TP9, and TP10, while the other examines F3, F4, C3, and C4.

In summary, the 19-channel configuration (Fp1, Fp2, F7, F3, Fz, F4, F8, T3, C3, Cz, C4, T4, T5, P3, Pz, P4, T6, O1, and O2) is commonly used among single-electrode setups. For bipolar electrode channels, besides 23 configurations (F8-F4, F7-F3, F4-C4, F3-C3, F4-Fz, Fz-Cz, F3-Fz, T4-C4, T3-C3, C4-Cz, C3-Cz, Cz-Pz, C4-P4, C3-P3, T4-T6, T3-T5, P4-Pz, P3-Pz, T6-O2, T5-O1, P4-O2, P3-O1, and O1-O2), no other bipolar electrode configurations were identified in LC-I.

- *Literature Collection II:*

EEG dataset channel descriptions from 39 articles have varied counts: 4, 8, 16, 19, 20, 21, 23, 32, and 64. Notably, 13 articles favored the 19-channel configuration, while 4 focused on 64 channels. Counts for datasets with 4, 8, 16, 20, 21, and 32 channels were 1, 1, 4, 2, 9, and 4 articles, respectively. Furthermore, one article involves 23-channel signals across two datasets. The 19-channel configuration ranks first, comprising (13+9) out of 39 articles (56.41%), with channel names consistent with those described in LC-I. However, LC-II contains no studies on bipolar electrode channels.

The pie chart ratio on the right in Figure 7 illustrates the proportions of articles based on different combinations of channel numbers in LC-II. Combined with the data from LC-I, the 19-channel combination stands out in terms of its application proportion across all articles. In practice, if devices with fewer channels are used, the 19-channel option should be considered first.

- ***Reference-style brief summary***

Similarities: The 19-channel data acquisition method has consistently received significant attention. Differences: In LC-II, the dual-electrode EEG acquisition method is not given as much emphasis. Connections: The number of channels in EEG acquisition devices varies considerably. Fewer channels focus on brain regions associated with cognitive function, while more channels provide broader coverage for comprehensive data collection. The 19-channel configuration is preferred for its balanced coverage and moderate channel count. Additionally, the inclusion of reference electrodes has also been recognized as beneficial.

### 3.3 Experimental protocol

EEG signal collection from patients and controls must specify scenarios for clarity. Due to physiological and non-physiological artifacts, raw signals need preprocessing for clean EEG data. This section outlines the protocol in two parts: paradigm types and preprocessing, based on literature.

#### 3.3.1 Paradigm types

- *Literature Collection I:*

Designing paradigms for patient-centered EEG studies requires careful consideration of participant states, experiment tolerance, and personal and environmental factors. Among the 92 articles, paradigms mainly focus on resting states: 9 for eyes-open, 38 for eyes-closed, 24 for alternating states, and 23 for other types (not mutually exclusive with the first three categories). Other paradigms include TMS (Kayasandik et al., 2022; Ferreri et al., 2021), cognitive ability tests (Ho et al., 2021a; Kim S.-K. et al., 2023), memory tests (Komolovaitė et al., 2022; Cicalese et al., 2020), stimulus induction (David et al., 2023; Tülay et al., 2020), image evocation (Seo et al., 2020; Güntekin et al., 2019), and fixation tasks (Kim et al., 2018; Moghadami et al., 2021). If possible, EEG can be collected simultaneously during memory and cognitive ability testing.

The duration for collecting individual trials is at least 8 s (Fouad and Labib, 2023), with most trials lasting 5 min. The length of EEG signal is generally 30 min. When collecting EEG from AD patients, it is essential to consider the rationality of paradigms, participants' adaptability, and potential issues. When recording the eyes-closed resting state EEG, the presence of a professional is necessary. If a participant shows signs of drowsiness, they must be promptly awakened (e.g., by calling them or other alerting cues) to eliminate interference.

- *Literature Collection II:*

In LC-II, data collection paradigms primarily focus on resting states, introducing 38 datasets from 38 articles. The eyes-closed resting state paradigm involves 26 articles, while 4 articles (Nour et al., 2024; Kim S.-K. et al., 2024; Wang et al., 2024) pertain to alternating eyes-open and eyes-closed paradigms. A dataset with part subjects in a resting state with their eyes open and part closed as a paradigm (Lee et al., 2025). Additionally, 5 articles (Jang et al., 2024; Meghdadi et al., 2024; Kim S.-H. et al., 2024) employed datasets designed with other tasks such as memory, oddball paradigm, taste stimuli (Olcay et al., 2025), video stimulation (Nguyen, 2025) and speech. One article combined the eyes-closed resting state with alternating eyes-open and eyes-closed conditions (Amato et al., 2024). The duration for a trial ranges from a minimum of 8 seconds to a maximum of 20 minutes, with most lasting under 10 min.

- ***Reference-style brief summary***

Similarities: The types of data collection paradigms predominantly focus on eyes-open and eyes-closed resting state conditions. Other paradigms, such as memory, cognitive tests, and stimulus responses, are also present. Differences: In LC-II, there is a noticeably lower proportion of articles employing paradigms other than the resting state compared to LC-I. Additionally, the duration of EEG signal collection varies between the two literature sets. Connections: This may be attributed to the greater controllability of the resting state paradigm, which explains the popularity of datasets utilizing the eyes-closed resting state approach in both literature collections. While collection times differ, single-trial durations of approximately 5 minutes are commonly utilized in research.

#### 3.3.2 Preprocessing

For EEG preprocessing methods, this review consolidates the main artifact removal techniques employed in the LC-I and LC-II, along with the relevant citations, organized into Table 1. This is intended to facilitate researchers' in-depth understanding of areas of interest. The following will further analyze its application.

- *Literature Collection I:*

**Table 1:** The main EEG preprocessing methods in LC-I and LC-II and their literature citations.

**Preprocessing methods**	**Main funtions**	**Related literature**
Filtering (high-pass, low-pass, band-pass, notch filtering)	Remove high-frequency noise and low-frequency drift.	Ding et al., 2022; Kopčanová et al., 2024; Fan et al., 2018; Tzimourta et al., 2019; Kayasandik et al., 2022; Zhao et al., 2019; Siuly et al., 2024; Nour et al., 2024; Rostamikia et al., 2024; Kim S.-K. et al., 2024; Yu et al., 2024; Yan et al., 2024; Puri et al., 2024; Mohammed, 2024; Goerttler et al., 2024; Gopu et al., 2024; Jang et al., 2024; Amato et al., 2024; Zheng et al., 2024b; Meghdadi et al., 2024; Lal et al., 2024; Simmatis et al., 2025; Cao et al., 2025; Olcay et al., 2025; Jain and Srivastava, 2025; Lee et al., 2025; Wang et al., 2025; Stefanou et al., 2025; Hachamnia et al., 2025; Ge et al., 2025; Acharya et al., 2025
Independent Component Analysis (ICA) and its improved algorithm	Remove artifacts from physiological signals and other sources.	Ge et al., 2020; Jesus et al., 2021; Lopes et al., 2023; Siuly et al., 2024; Kim S.-K. et al., 2024; Yan et al., 2024; Gopu et al., 2024; Jang et al., 2024; Amato et al., 2024; Zheng et al., 2024b; Meghdadi et al., 2024; Lal et al., 2024; Sreedhar et al., 2025; Puri et al., 2025; Cao et al., 2025; Olcay et al., 2025; Jain and Srivastava, 2025; Stefanou et al., 2025; Hachamnia et al., 2025; Ge et al., 2025; Acharya et al., 2025
Visual inspection	Manually remove obvious abnormal pattern signals.	Nobukawa et al., 2020; Ruiz-Gómez et al., 2018a; Ge et al., 2020; Güntekin et al., 2019; Tăuţan et al., 2023; Cejnek et al., 2021; Amezquita-Sanchez et al., 2019; Ruiz-Gómez et al., 2018b; Song et al., 2021; Tucci et al., 2023
Interpolation algorithms	Filling in missing or corrupted channel data.	Ding et al., 2022; Ge et al., 2020; Lassi et al., 2023; Kopčanová et al., 2024; Tăuţan et al., 2022b
Artifact Subspace Reconstruction (ASR)	Reconstruct the signal to adaptively remove artifacts.	Si et al., 2023; Zheng et al., 2023; Rostamikia et al., 2024; Puri et al., 2024; Zheng et al., 2024b; Lal et al., 2024; Puri et al., 2025; Wang et al., 2025; Stefanou et al., 2025; Hachamnia et al., 2025; Acharya et al., 2025
Program plugin toolbox (EEGLAB, Brain Vision Analyzer, etc.)	Includes the above functions. More convenient to remove artifacts.	Andrade et al., 2023; Tülay et al., 2020; Meghdadi et al., 2021b; Lassi et al., 2023; Tavares et al., 2019; Sibilano et al., 2023; Ding et al., 2022; Kopčanová et al., 2024; Song et al., 2022; Farina et al., 2020

Artifacts such as electrooculographic (EOG), electromyographic (EMG), electrocardiographic (ECG) artifacts, and power line interference are unavoidable during data collection. In LC-I, 76 articles provided methods for artifact removal, while the remaining articles either did not specify methods or indicated that publicly processed data was used.

First, downsampling reduces the amount of data (Ge et al., 2020), although this step is not essential. To prevent interference from EMG and other signals, a bandpass filter (zero-phase finite impulse response (FIR), Butterworth, or Chebyshev II) is applied to retain the information of interest. Some authors utilize separate high-pass and low-pass filters for the same purpose. Notch filters are essential for eliminating power line frequency interference. However, the EEG signal may still contain artifacts from EOG, ECG, EMG, and other sources. Independent component analysis (ICA) helps remove these artifacts by discarding relevant components. Artifact removal is not limited to these methods. Professionals can also manually remove some artifacts through visual inspection. If segments or channels have unavailable data, interpolation methods such as spherical interpolation and cubic interpolation can ensure data integrity. Additionally, some studies re-reference signals after preliminary artifact removal. The literature also discusses other artifact processing methods such as wavelet transform (Vicchietti et al., 2023; Li et al., 2021), Fourier transform (He et al., 2023; Gunawardena et al., 2023), ICA, blind source separation (Chamoli, 2021), and the artifact subspace reconstruction (ASR) algorithm, often used in combination (Kim N. H. et al., 2023). Furthermore, various software programs and plugins, such as Brain Vision Analyzer and EEGLAB are widely utilized.

Preprocessing methods primarily use traditional techniques, though automated approaches like EEGLAB are increasingly popular. Less commonly employed automated data-driven methods, such as the Autoreject algorithm (Perez-Valero et al., 2022), have also been applied in EEG data processing for AD. Regardless of the approach, the objective is to extract cleaner EEG information.

- *Literature Collection II:*

The LC-II does not present novel insights. Authors typically use filters to eliminate artifacts. ICA and its improved methods are widely employed. Among these, the ASR method remains frequently utilized. Automated software programs for artifact removal primarily focus on EEGLAB. Interpolation correction methods are still used for erroneous EEG data (Yan et al., 2024; Simmatis et al., 2025). Although literature on expert visual inspection is limited (Babiloni et al., 2024), it is still practiced. Furthermore, the Savitzky-Golay filter (Ohal and Mantri, 2024) has been employed for EEG data processing.

The preprocessing steps for AD-related EEG are consistent with those of a standard BCI system, showing no significant differences. In addition to traditional methods, several novel approaches have emerged in recent years. For instance, the Harvard Automated Processing Pipeline for EEG (HAPPE) is a processing method that integrates multiple algorithms, involving a two-step ICA approach. This includes wavelet-enhanced ICA for artifact removal and ICA decomposition, combined with a multi-artifact suppression algorithm to enhance the effectiveness of artifact removal. There is anticipation for the introduction of more innovative, integrated automatic artifact removal algorithms.

### 3.4 Characteristic form

Dementia patients have different EEG signals from healthy individuals due to nerve damage. The literature emphasizes that EEG features can be used as biomarkers for AD analysis. This section reviews AD data feature forms from the perspectives of EEG rhythms and features.

#### 3.4.1 EEG rhythms

- *Literature Collection I:*

EEG is typically divided into several frequency bands based on biological foundations to examine their effects. This approach is still adopted in research on biomarkers for AD.

**Figure 8:**
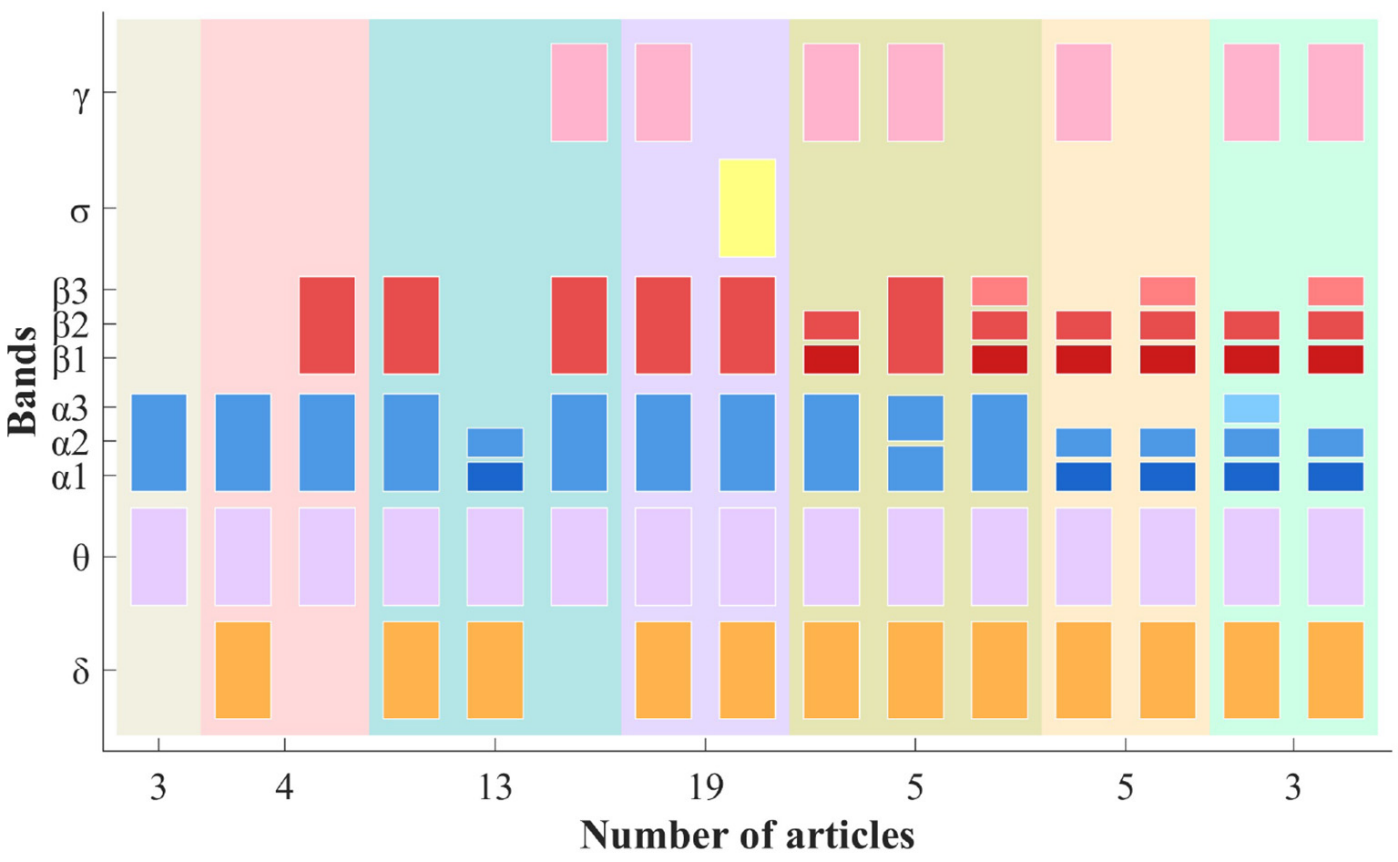
The main frequency band divisions and their corresponding number of articles in LC-I [the same background color indicates that the articles involve the same number of bands, while the horizontal axis represents the total number of articles. Additionally, one paper includes several cross-frequency data: δ (0.5–4 Hz), θ (4–8 Hz), α (8–12 Hz), low α (8–10 Hz), high α (10–12 Hz), β (12–30 Hz), δ-β (0.5–30 Hz), θ-β (4–30 Hz), and low γ (30–45 Hz)].

In 53 articles, data underwent frequency band decomposition, while the remaining articles used full-band data or did not specify their methodology. The combinations are categorized into eight cases based on the frequency bands counts (B.C.): 2, 3, 4, 5, 6, 7, 8, and 9. While the bands are consistent, there are deviations in the numerical values of the filtering frequencies. The upper limit of the γ band is 45 Hz or higher. Meanwhile, a single band can be divided equally into two or three levels. This analysis does not delve deeply into specific frequencies. Figure 8 shows the distribution of main frequency band divisions involved in LC-I.

From Figure 8, research on EEG rhythms in AD primarily focuses on the five common frequency bands. The θ rhythm is consistently present across all combinations, while the sigma rhythm (12.5–15.5 Hz), close to the β range, has been considered only once. In detailed band combinations, α and β are preferred for classification. In addition, the full-band data is also considered (Klepl et al., 2022; Tsolakis et al., 2022). Furthermore, frequency band ratios can serve as a standard for EEG rhythm analysis in AD.

- *Literature Collection II:*

In LC-II, 18 articles discuss EEG data filtering, while 5 articles provide filtering ranges without detailing band name: 0.5–45 Hz (Rostamikia et al., 2024; Lal et al., 2024), 0.1–41 Hz (Yu et al., 2024), 0.1–100 Hz (Yan et al., 2024), and 8–30 Hz (Mohammed, 2024). Furthermore, the EEG frequency band divisions in LC-I already cover the main cases. The more detailed band divisions in LC-II are encompassed within LC-I. To highlight recent areas of interest, this section will present a table showcasing the combinations of frequency band divisions in LC-II along with the corresponding number of articles. The frequency band combinations for the related article counts (A.C.) are detailed in Table 2.

**Table 2:** Frequency band divisions in Literature Collection II.

**No**.	**B.C**.	**A.C**.	**Band divisions condition**
1	4	7	δ, θ, α, and β
			δ, θ, α, and 0.5–45 Hz (or 1–40 Hz)
2	5	10	δ, θ, α, β, and γ;
			δ, θ, α1, α2, and α3 (Babiloni et al., 2024)
3	6	4	δ, θ, α, β1, β2, and γ;
			δ, θ, α, β, low γ, and high γ (Kim S.-H. et al., 2024)
4	8	1	δ (1–3 Hz), slow θ (3–5 Hz), θ (3–7 Hz), slow α (8–10 Hz), α (8–13 Hz), slow β (13–20 Hz), β (13–30 Hz), and γ (25–40 Hz)

Similar to LC-I, there is a greater emphasis on 5 frequency band combinations (Nour et al., 2024; Babiloni et al., 2024; Zheng et al., 2024b). Additionally, traditional band data appears in 9 out of 10 articles in LC-II.

- ***Reference-style brief summary***

Similarities: In both literature sets, the frequency bands consistently include theta and alpha bands, with the α featuring more detailed subdivisions. Differences: LC-I considers the σ rhythm, which is absent in LC-II. However, LC-II does include whole-band divisions and does not perform additional rhythmic segmentation of the data. Connections: The traditional frequency band divisions are still frequently applied in the articles. Nevertheless, more detailed divisions may be introduced for targeted studies on specific frequency band effects, particularly concerning the α. Meanwhile, the influence of whole-band information in research should not be overlooked.

#### 3.4.2 EEG features

Researchers use EEG features as biomarkers for AD to achieve discriminative identification. Different domain-specific features or new feature analysis methods are employed to describe or capture AD-related signature information. The extracted features are often categorized into four types: time domain, frequency domain, time-frequency domain, and spatial domain (correlation).

- *Literature Collection I:*

Based on the EEG features discussed in LC-I, this section provides a concise overview of the main trends in analysis across four fields and lists the key features types involved.

**Figure 9:**
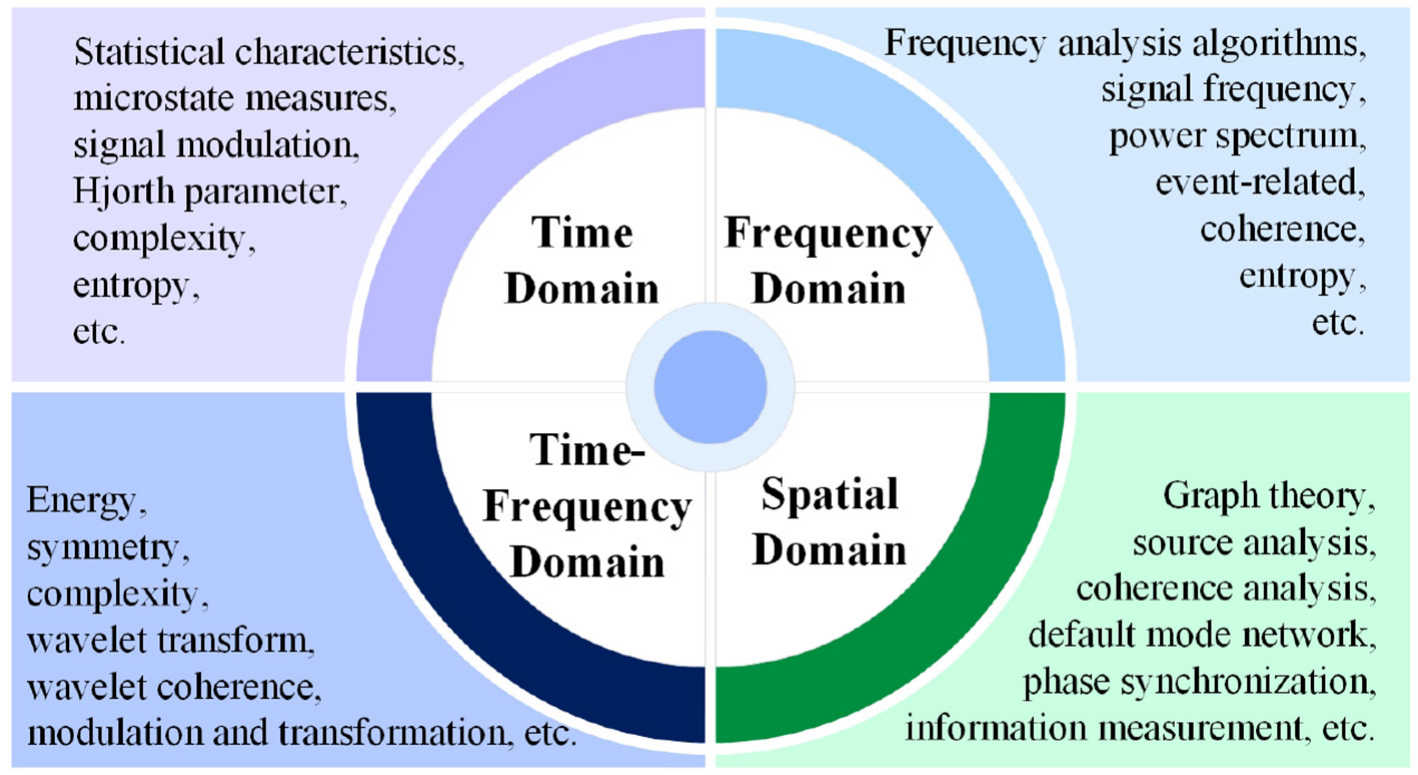
Distribution of major types of EEG biomarkers for AD in LC-I.

Figure 9 shows the main distribution types of features from different domains. The specific features involved are detailed in the Supplementary material.

**Time domain:** The primary focus is on statistical feature extraction, with methods also capturing nonlinear features from time series data and analyzing microstate information (Lassi et al., 2023; Jiao et al., 2023). **Frequency domain:** Analysis emphasizes power and spectral characteristics, along with energy-related features. **Time-frequency domain:** Wavelet transform is dominant, with its derived features actively analyzed. **Spatial domain:** This field focuses on network analysis, encompassing functional connectivity, effective connectivity, and graph analysis. Additionally, analyses of coherence are also of interest.

The exploration of EEG biomarkers for AD extends beyond the aforementioned features to include hybrid features. For example, other domain features (such as variance Ge et al., 2020, Pearson correlation coefficient, interquartile range, and Hoeffding's D metric) can be extracted after processing EEG signals in the time-frequency domain. Researchers are also designing new EEG features to analyze AD. Dogan et al. (2023) extracted a novel primate brain pattern (PBP) that considers brain regions like visual and motor areas as segments (vertices) for functional analysis, with directed edges representing interconnected neuronal connections. PBP serves as an innovative graph-based local texture feature extractor. Gunawardena et al. (2023) developed a model using Isomap and Gaussian Process Latent Variable Model (Isomap-GPLVM), which produces a kernel (dis)similarity matrix used as a metric for linear and nonlinear functional connectivity between EEG channels. For source localization analysis, the eLORETA software has been widely used to trace relationships between brain channels (Vecchio et al., 2020; San-Martin et al., 2021; Del Percio et al., 2019; Aoki et al., 2023).

**Figure 10:**
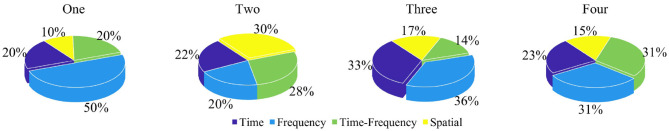
Domain application ratios among the groups (One, Two, Three, and Four classes) in LC-I.

To highlight the significance of groups and features in this research, this review reselected articles that apply both, totaling 73 datasets. These comprise one class (5 datasets), two classes (34 datasets), three classes (25 datasets), and four classes (9 datasets). To illustrate research trends in feature analysis, the application frequencies of different feature domains within these groups will be visualized proportionally. It's important to note the number of different domains recorded within the same group. For innovative features, the domains considered will also be counted as instances of domain application. Figure 10 illustrates the domain application ratios among the four groups in LC-I. Among them, the emphasis on time domain features remains relatively stable.

- *Literature Collection II:*

In LC-II, many features overlaps with those in LC-I. In the time domain, statistical features and entropy features are prominent, along with various fractal dimensions, such as Katz, Petrosian, and Higuchi based on fractal geometry. One article focuses on Hilbert-Huang features (Gopu et al., 2024) to analyze EEG. Frequency domain features concentrate on PSD and power (including their ratios). Research on time-frequency domain features is limited (Kim S.-K. et al., 2024; Jain and Srivastava, 2025), mainly addressing the classification of total activity, phase-locked activity, non-phase-locked activity, FFT, and synchrosqueezing transform. In the spatial domain, studies often examine coherence and correlation (Yu et al., 2024; Amato et al., 2024; Jiang et al., 2025; Cao et al., 2025). Researchers also explored EEG connectivity across 246 cortical regions (Youn et al., 2024). The Second Order Difference Plot (SODP) is also considered in EEG analysis of AD (Wang et al., 2024).

Furthermore, the design of new features remains a research direction for exploring AD biomarkers. In LC-II, Mohammed (2024) utilized Mel frequency cepstral coefficients (MFCC) features for EEG analysis related to AD. Meanwhile, Goerttler et al. (2024) introduced a new feature extraction tool that includes windowing, PSD features, temporal pooling, and spatial pooling, which examines EEG spatial information by altering dimensional data. Zheng et al. (2024b) developed and calculated the Multi-Threshold Recurrence Rate Plot (MTRRP) metrics, including recurrence complexity, recurrence hurst, and recurrence rate gradient to investigate AD biomarkers.

**Figure 11:**
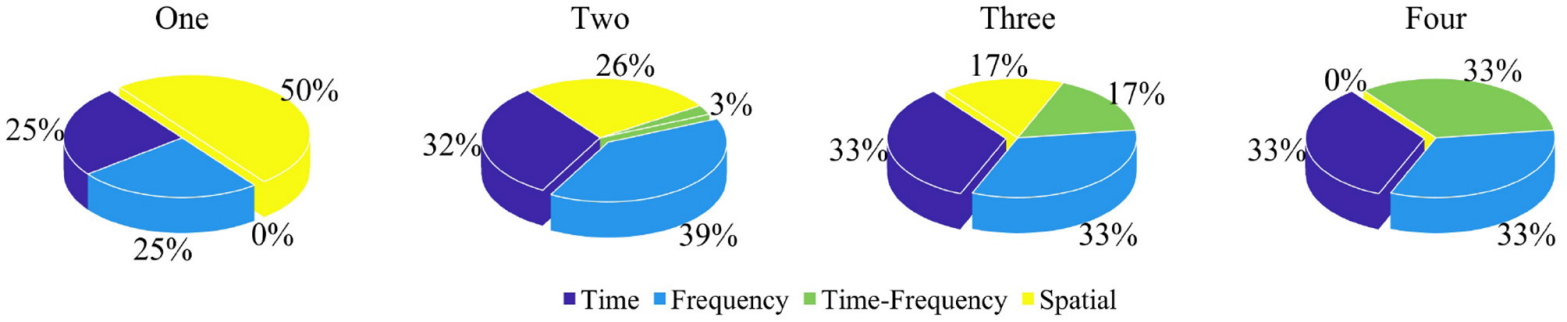
Domain application ratios among the groups (One, Two, Three, and Four classes) in LC-II.

Similarly, the proportions of feature domains in groups of LC-II are analyzed. A total of 40 datasets are involved: one class (2 datasets), two classes (19 datasets), three classes (18 datasets), and four classes (1 dataset). Figure 11 visualizes the application ratios of feature domains among the four groups in LC-II. The importance of feature domains varies, with the proportions of time domain and frequency domain applications being relatively stable.

**Figure 12:**
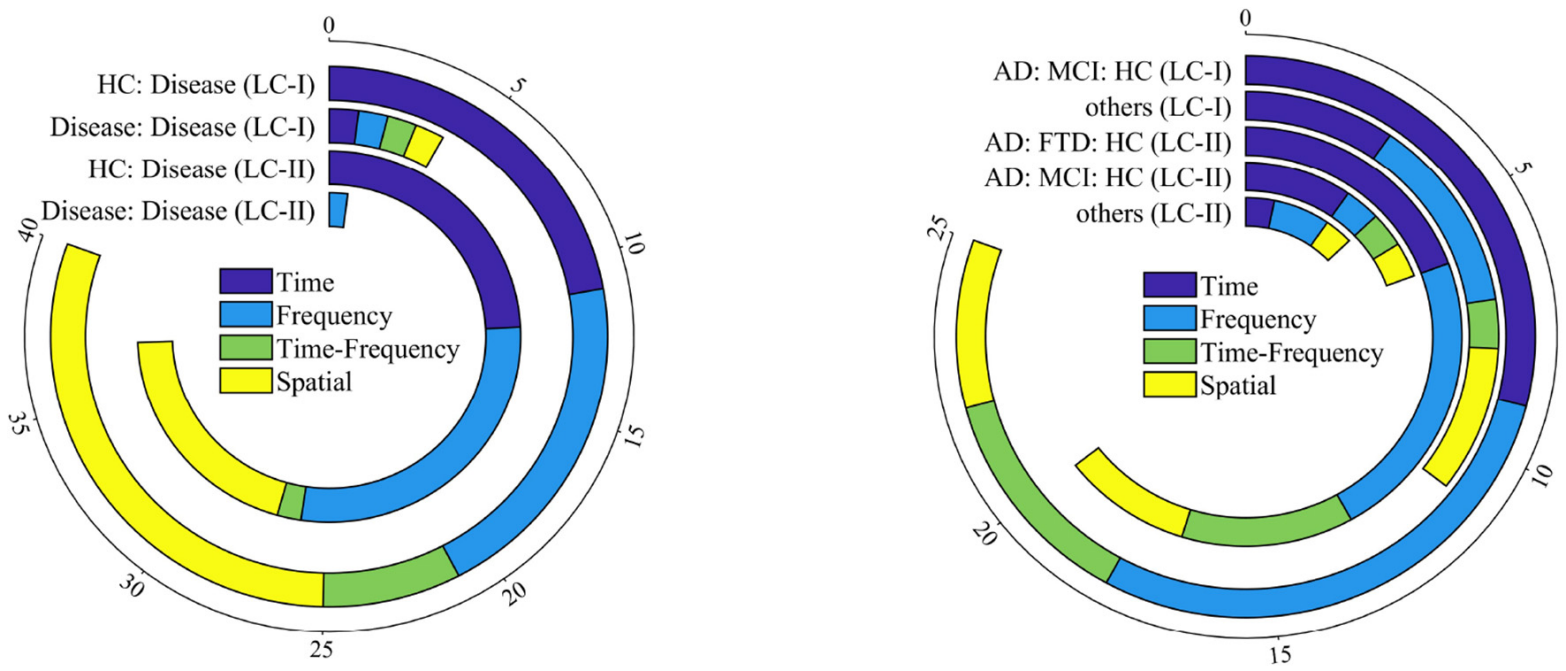
Domain application numbers on Two and Three classes (from left to right) in LC-I and LC-II.

To clearly illustrate the application of features within specific categories, this section visualizes the frequency of feature applications for the two most utilized groups (Two and Three classes) by a stacked ring chart in Figure 12. It is significant to note that the displayed category combinations represent those with higher application frequencies. In both the Two and Three classes scenarios, the chart indicates that time domain and frequency domain features are among the most frequently used. Feature extraction is often regarded as the foundational exploration of biomarkers. An increasing number of researchers are turning to feature innovation to uncover significant information. For instance, combining low-frequency and high-frequency scale maps to obtain a two-dimensional relative energy distribution vector, or combining rational and binary filters to create new wavelet filters, are examples of innovative approaches. Additionally, there are innovative methods for extracting signal correlation features based on graph models. To better capture useful information, the integration of convolutional networks and temporal networks has also been applied. More research focuses on exploring spatial information relationships within temporal and frequency domain information to associate them with neurodegenerative patterns.

- ***Reference-style brief summary***

Similarities: The characteristics of traditional methods have not been abandoned in the exploration of EEG in AD. Differences: Various innovative feature constructions are continually emerging in the EEG analysis of AD. Connections: From traditional to innovative approaches, the exploration methods in both literature sets remain consistent. The goal is to develop EEG biomarkers that are more relevant to AD. However, there is no unique representation of effective features. Therefore, researchers continue to explore new features.

### 3.5 Analytical methods

In this manuscript, the term “analysis methods” refers to a broad concept that encompasses multiple steps, including models aimed at only feature extraction, classification models, evaluation models, etc. In contrast, “classification methods” specifically refers to algorithmic approaches designed for the purpose of classification.

After EEG feature extraction, classifier selection and design are the primary focus in AD recognition analysis. This section primarily summarizes classifiers from the perspectives of machine learning and deep neural learning, with brief mentions of other analytical approaches. Lastly, a concise summary of the performance evaluation will be provided.

- *Literature Collection I:*

**Figure 13:**
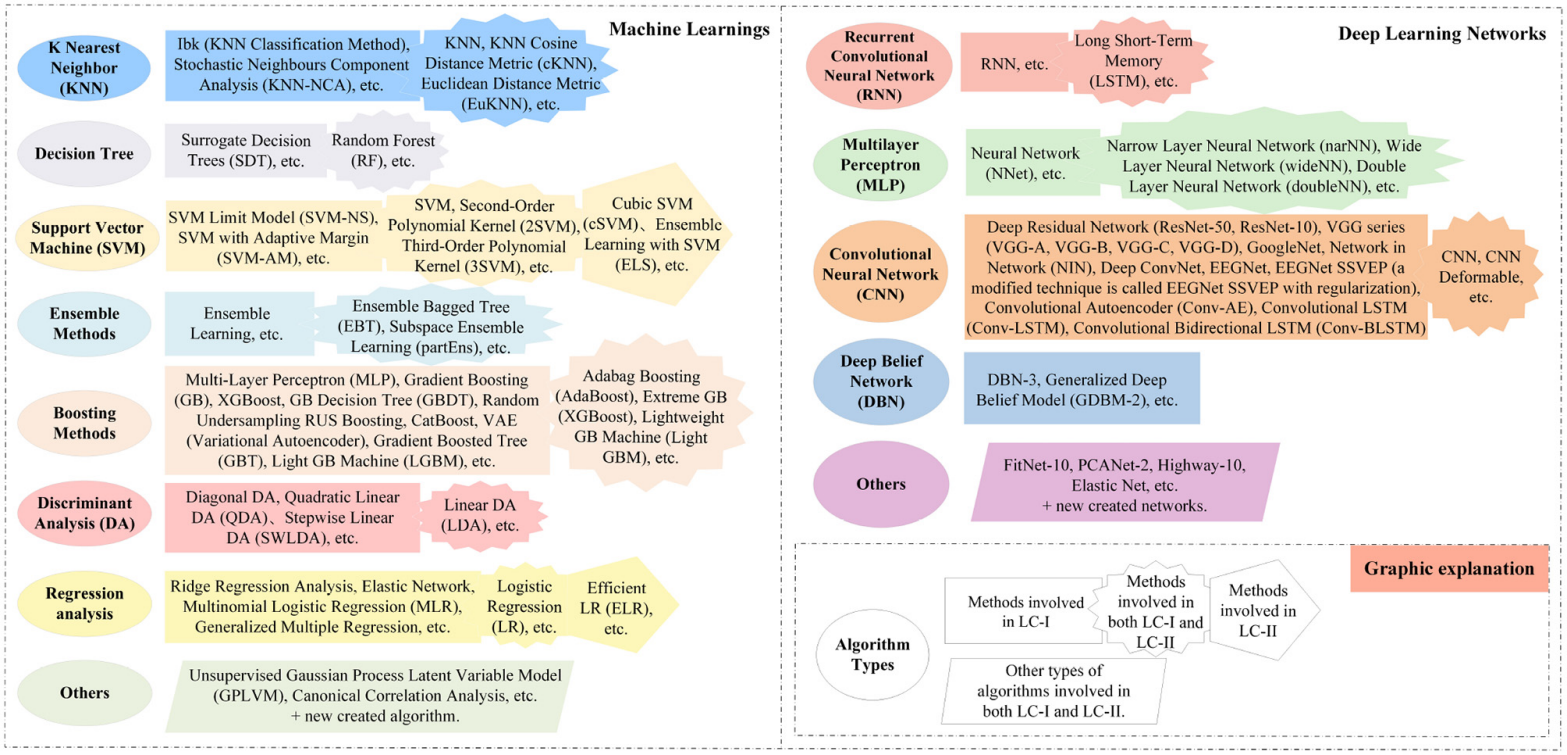
A partial summary of the main recognition algorithms involved in LC-I and LC-II.

Figure 13 shows some algorithms for EEG-based AD recognition in two literature sets.

#### 3.5.1 Classifiers

The main identification methods in LC-I are listed in Figure 10. In addition, some innovative algorithms and statistical analysis are applied in some articles.

Researchers are actively exploring and creating new networks for EEG-based AD identification. For example a new multi-feature fusion learning architecture of ViT and CNN (Chen et al., 2023), a multi-channel EEG AD recognition network improved based on the CNN-LSTM model (DeepADNet) (Ho et al., 2021a), a fusion network of ResNet-18 and RNN (Sibilano et al., 2023), a discriminative subspace low-rank representation learning algorithm (DSLRR) (Tang et al., 2022), a fusion learning process weight model (EWGLO-OTALSTM) (Bhendare and Dewangan, 2023), a frequency-based multi-layer network (FMN) (Si et al., 2023), a discriminative version of Contractive Slab and Spike Convolutional Deep Boltzmann Machine (DCssCDBM) (Bi and Wang, 2019), error and learning-based novelty detection (ELBND) (Cejnek et al., 2021), improved deep pyramid convolutional neural network (DPCNN) model (Xia et al., 2023), sandpiper-based recurrent neural system (SbRNS) (Swarnalatha, 2023), adaptive gated graph convolutional network (AGGCN) (Klepl et al., 2022), etc. Innovative models mainly focus on model structure (integration among models), enhancing capture information (extracting more domain features), and model optimization, etc. Regardless of the approach, the goal is to improve the identification of AD.

To analyze the differences in EEG signals among AD patients groups, statistical tests (such as t-tests, ANOVA, Mauchly's test, Duncan's test, Grubbs' test, Levene's test, Kolmogorov-Smirnov test, and Shapiro-Wilk test) are actively employed alongside other analytical approaches. Statistical analysis methods (Vicchietti et al., 2023; Tylová et al., 2018; Puri et al., 2023) not only apply to EEG analysis but also consider other non-quantitative information (Waser et al., 2019) (such as age, gender, and years of education) that may impact AD. Additionally, Lassi et al. (2023) utilized the ATN (amyloid, tau, neurodegeneration) system (Ebenau et al., 2020) to analyze the topological states of EEG microstates, examining the differences in AD across groups.

- *Literature Collection II:*

Figure 10 also displays the main learning methods in LC-II and lists the common elements from the two collections separately.

Furthermore, new model designs continue to advance in AD classification. Nour et al. (2024) proposed an ensemble learning model by Deep Ensemble Learning (DEL) and 2-dimensional Convolutional Neural Networks (2D-CNN), demonstrating high accuracy in AD classification for the first time. Puri et al. (2024) introduced the Rational Dyadic Biorthogonal Wavelet Filter Banks (RDBWFBs) model, which outperformed existing wavelet filters on effectiveness. Both approaches aim to capture more distinguishing information between AD and other groups for improved classification. Wang et al. (2024) proposed an Improved Artificial Fish-Swarm-Genetic Algorithm (IAFS-GA) model that optimizes channel combinations by integrating the Artificial Fish-Swarm Algorithm (AFSA) and Genetic Algorithm (GA), enhancing classification performance. Additionally, (Bunterngchit et al. 2024) integrated Attention into the CNN-LSTM model to improve focus on key information, achieving higher recognition accuracy. Meanwhile, transformer combination models (Stefanou et al., 2025; Acharya et al., 2025) and multi-model integration models (Hachamnia et al., 2025) are also frequently proposed.

Statistical methods such as p-value calculation, eLORETA analysis, and PCA are still significant in EEG analysis for AD.

The design of classifiers also adheres to the principle of maximizing the discrimination of effective EEG information. To focus on important information, attention mechanisms have been integrated with convolutional neural networks and temporal networks to achieve high-performance classification. Additionally, some predictive regression models combined with complex networks are used for disease prediction analysis of patients EEG. Methods such as normalization, correlation analysis, and dimensional transformation models have also been applied in the innovation of signal features and classification models.

- ***Reference-style brief summary***

Similarities and Differences: In both literature sets, classifiers include machine learning and deep learning models, with similarities and differences aligned with the underlying principles of model construction. Regardless of the model, the primary goal is to achieve the classification of AD. Commonly used models include SVM, convolutional models, and variations of time series models. Connections: A comprehensive analysis of literature collections and Figure 10 show that machine learning dominates AD recognition, with significant attention on SVM and its variants. CNN is a key focus in deep learning models, while various forms of ANN and RNN variants effectively support AD recognition. In LC-II, statistical methods primarily assess the significance of features, channels, and classification performance, whereas LC-I often utilizes statistical methods as standalone discrimination techniques. Importantly, statistical testing approaches complement rather than contradict learning models.

#### 3.5.2 Performance evaluation

In analyzing the EEG of individuals with cognitive impairments, certain quantitative metrics are essential for assessing performance of feature extraction and classification methods for EEG analysis in dementia.

- *Literature Collection I:*

The authors employed various indicators to evaluate recognition performance and analyze data relationships, including accuracy, precision, recall, F1 score, specificity (true negative rate), sensitivity (true positive rate), confusion matrix, F-measure, G-mean, Jaccard (Tang et al., 2022), AUC, ROC, HTER mean, HTER standard deviation (Gore and Rathi, 2022), positive predictive value (PPV), negative predictive value (NPV) (Laton et al., 2022), kappa statistic, and p-value.

- *Literature Collection II:*

The evaluation methods in LC-II resemble those in LC-I, still utilizing less common evaluation metrics (Siuly et al., 2024; Yan et al., 2024) such as Matthews Correlation Coefficient and Kappa coefficient.

The evaluation methods can be single or mixed, aimed at assessing disease recognition through EEG, similar to other performance evaluation methods. Cross-validation methods are widely operated to enhance the model's generalization ability, ensuring more stable and reliable performance on unseen data.

In numerous studies, accuracy is the primary metric for evaluating performance. Follow-up analyses of specific subject groups commonly use before-after comparisons, including protein testing. Multi-category studies typically employ learning methods for classification. Five 2D-CNN models achieved an average accuracy of 97.9% for AD vs. HC (Nour et al., 2024). The innovative LSTM model reached 97% in three-class recognition of HC vs. AD1 vs. AD2 (Siuly et al., 2024). A review of the LC-II indicates a trend toward distinguishing between diseased and non-diseased groups, further subdividing the diseased group. SVMs attained recognition rates of 87.8% and 93.5% in AD vs. FTD and AD vs. HC, respectively (Rostamikia et al., 2024). More discriminative methods in dementia EEG have shown performances exceeding 85% in two and three-group comparisons, while rest-EEG accuracy reaches 67.24% in four classes with AD (Kim S.-K. et al., 2024).

It is worth emphasizing once again that most studies consider cross-validation as the primary choice to enhance recognition performance. Researchers interested in the application scenarios of cross-validation methods and the specific parameters used can refer to the following literature (Tzimourta et al., 2019; Kayasandik et al., 2022; Komolovaitė et al., 2022; Cicalese et al., 2020; Tăuţan et al., 2022a; Ge et al., 2020; Fan et al., 2018; Zhao et al., 2019; Vicchietti et al., 2023; Gunawardena et al., 2023; Zheng et al., 2023; Tavares et al., 2019; Song et al., 2022; Perez-Valero et al., 2022; Klepl et al., 2022; Xia et al., 2023; Calub et al., 2023; Parihar and Swami, 2023; Gkenios et al., 2022; Ranjan and Kumar, 2023; Zheng et al., 2024a; Simmatis et al., 2025; Jiang et al., 2025; Sreedhar et al., 2025; Nguyen, 2025; Puri et al., 2025; Olcay et al., 2025; Jain and Srivastava, 2025; Lee et al., 2025; Wang et al., 2025; Hachamnia et al., 2025; Ge et al., 2025).

## 4 Discussion of results

The previous section provided a brief summary of important data from the literature regarding key structures. To present the entire analysis process of AD-related research more clearly, the Table 3 summarizes typical literature from 2018 to 2025 (one paper per year from 2018 to 2023, and two papers per year for 2024 and 2025). The main content includes: Groups, Paradigms, Channels, Features, Main Analytical Methods (data-level analysis and classification methods), Main Conclusions (identification effectiveness and findings), and Prospects. This table allows for a clearer articulation of the main conclusions in the “Reference-style Brief Summary.”

**Table 3:** Important landmark contents in typical references from different years (Years, Groups, Para. (Paradigms), C. (Channels), Features, Main Analytical Methods, Main Conclusions, Prospects).

**Years**	**Groups**	**Para**.	**C**.	**Features**	**Main analytical methods**	**Main conclusions**	**Prospects**
2018 (Fan et al., 2018)	Four classes (HC: AD1: AD2: AD3)	Resting (close eyes)	19	0.05–70Hz. Features: Multiscale Entropy Analysis (MSE).	Analysis: canonical correlation analysis (CCA). Classifier: logistic regression (LR).	**Results:** 80%. **Findings:** The long-term complexity of EEG decreases with the severity of AD, while the temporal and parieto-occipital brain regions are more effective in distinguishing severe AD patients from HC.	Integrate the exploratory patterns of AD research, including EEG complexity, synchrony, and functional connectivity.
2019 (Yu et al., 2019)	Two classes: (HC: AD)	Resting (close and open eyes)	16	δ, θ, α, and γ. Features: Phase synchronization index (PSI)	Takagi-Sugeno-Kang (N-TSK).	**Results:** the highest: 97.3% (close eyes), 94.78% (open eyes). **Findings:** Local efficiency and clustering coefficient are most effective in AD identification.	Combine semi-supervised or unsupervised learning with complex network methods to reduce their dependence on data label acquisition.
2020 (Farina et al., 2020)	Three classes: (HC: MCI: AD)	Resting (close and open eyes)	30	δ, θ, α1, α2, β1, β2, and γ. Features: absolute power, relative power, and weighted phase lag index.	A penalized logistic regression algorithm with a well-established regularization technique known as the Elastic Net.	**Results:** sMRI outperforms resting-state EEG in classifying AD (AUCs = 1.00 vs. 0.76). Both EEG and sMRI show only moderate performance in distinguishing MCI from healthy aging. **Findings:** AD status is linked to increased connectivity between left temporoparietal electrodes in the theta and delta.	Prioritize longitudinal data collection to track the predictive ability of resting-state EEG features for AD and MCI, as well as to differentiate AD from other forms of dementia.
2021 (Li et al., 2021)	Two classes: (HC: AD)	Resting (close eyes)	16	δ, θ, α, and β. Features: relative power and PSD.	Analysis: variational auto-encoder. Classifier: neural dynamic modeling, representational modeling, and Takagi-Sugeno-Kang (TSK).	**Results:** 98.10%. **Findings:** An increase in theta activity and a decrease in alpha activity may be early signs of cognitive decline in AD.	Expand the dataset and integrate complex network methods with machine learning techniques to design a more accurate EEG recognition model for AD.
2022 (Tang et al., 2022)	Three classes: (HC: MCI: AD)	Resting (close eyes)	19	Fourier coefficients	Discriminant subspace low-rank representation learning algorithm (DSLRR).	**Results:** more than 97.26% (AD vs. HC, MCI vs. HC, HC vs. MCI & AD). **Findings:** The model effectively ignores redundant information and extracts structural knowledge and manifold information from EEG, enabling efficient identification of MCI and AD in HC.	The model expands to a multi-feature scenario, enriching the AD recognition system. It will also leverage transfer learning to improve algorithm generalization.
2023 (Si et al., 2023)	Three classes: (HC: FTD: AD)	Resting (close eyes)	19	δ, θ, α, sigma, and β. Features: Phase lock value (PLV), intra-frequency coupling, cross-frequency coupling, and graph features.	Analysis: ANOVA, F-score, multiple linear regression. Classifier: frequency-based multilayer resting-state EEG networks (12 machine learning).	**Results:** the predicted and actual measured scores were indeed significantly correlated with each other (r = 0.274, *p* = 0.036); 81.1%, 86.2%, 84.7% (AD vs. FTD, AD vs. HC, and FTD vs. HC). **Findings:** Compared to HC, AD shows stronger δ-α cross-coupling and weaker θ-sigma cross-coupling. Additionally, AD exhibits stronger δ-α and δ-β connectivity than FTD.	This research holds promise for clinical application by providing potential biomarkers that can distinguish between AD and FTD.
2024 (Nour et al., 2024)	Two classes: (HC: AD)	Resting (open and close eyes)	19	δ, θ, α, β, and γ. Features: generated by 2D-CNN.	Deep ensemble learning classification model (Consists of 5 2D-CNNs).	**Results:** 97.9%. **Findings:** EEG can identify specific bands oscillations associated with AD. This model achieves high recognition performance across the δ, θ, α, β, and γ.	The model can be applied to other neurological disorders in the future. Additionally, integrating multiple modalities will facilitate the development of deep learning models for diagnosing neurological diseases.
2024 (Siuly et al., 2024)	Three classes: (HC: AD1: AD2)	Resting (close eyes)	20	δ, θ, α, β, and γ. Features: mean, median, Lomb-Scargle periodogram Power Spectral Density (LPSD), and Welch's PSD (WPSD).	Analysis: One-way ANOVA. Classifier: feature-combined LSTM.	**Results:** more than 80% (β and γ); 95% (critical channels). **Findings:** 'Cz, F4, P4, T6, and Pz' in beta and gamma are the best EEG biomarkers for AD detection.	Model expansion is expected to significantly aid in the automatic diagnosis of various AD types.
2025 (Olcay et al., 2025)	Two classes: (HC: AD)	Olfactory stimulation	32	δ, θ, α, β, and γ. Features: continuous wavelet transform (CWT) scalogram, and the statistical distance of relative energy distribution (seven types).	Analysis: Fisher score (feature), and Spearman rank correlation. Classifier: Fisher linear discriminant (FLD), linear and nonlinear SVM, and k-nearest neighbor classifiers (kNN).	**Results:** 87.50%. **Findings:** AD patients exhibit changes in brain temporal organization after olfactory stimulation, especially in delta and alpha activity from Fp1 and beta activity from Pz. These temporal responses can help distinguish AD.	Explore new treatment strategies. In the future, combining olfactory tests with cognitive assessments may enhance diagnostic accuracy.
2025 (Sreedhar et al., 2025)	Three classes: (HC: FTD: AD)	Resting (close eyes)	32	0.5–45Hz	Fuzzy Recurrent Neural Network (Fuzzy RNN) based on Takagi-Sugeno-Kang (TSK).	**Results:** 98.82 (AD vs. HC), 98.42 (FTD vs. HC), and 97.82 (AD vs. FTD). **Findings:** Compared to other models, this one has stability and the ability to identify subtle patterns associated with cognitive impairment.	The robustness of the model in real clinical environments can be explored, and multimodal data sources can be integrated to develop a more comprehensive diagnostic approach.

In terms of groups, the analysis primarily consists of two-category and three-category approaches. In the paradigms, resting-state analysis is mainly employed. Channel configurations typically use a 19-channel layout, and features include traditional frequency bands as well as refined and innovative characteristics. The analytical methods focus on capturing information features from the biological perspective of EEG in AD, encompassing feature extraction and classification innovation, with identification performance exceeding 85%. The findings are primarily articulated from the perspectives of frequency bands obtained from the analysis (such as the alpha band and cross-coupling analysis), features (temporal and spatial information analyzed in greater detail due to their interpretability), and channels (increased analysis of the temporal and parietal lobes), among others. To achieve a more systematic understanding of the findings, this section will discuss some results.

AD is an irreversible neurodegenerative disorder, with treatments only delaying progression. Medical diagnosis often relies on invasive techniques or costly imaging methods. Given the correlation between EEG and brain activity, researchers are exploring the relationship between non-invasive EEG data and AD.

Because dementia severity is assessed by the clinical MMSE scale, the ambiguity of patient background information has no significant impact on the discussion of the results in this review. Numerous researchers have explored EEG recognition to distinguish AD patients from non-AD patients, investigating EEG rhythms, features, brain regions, and identification methods to find effective approaches. However, there is currently no consensus on systematic conclusions, as s different methods yield similar results but varying interpretations. This section will highlight prominent findings from the literature.

### 4.1 EEG biomarkers

This subsection will discuss the conclusions drawn from the literature on EEG-based AD identification.

#### 4.1.1 Paradigmatic form of EEG acquisition

In resting-state EEG analysis, the combined eyes-open and eyes-closed EEG recognition performance is inferior to individual states (Şeker et al., 2021). Eyes-open EEG is more effective at distinguishing patients from controls than eyes-closed resting-state EEG (Klepl et al., 2023). However, it is crucial to note that individual differences may affect the validity of the results. Further research should expand the dataset to minimize the impact of individual variability on the effectiveness of EEG in distinguishing AD during either eyes-open or eyes-closed resting-states. Additionally, EEG classification during memory encoding outperforms resting states (Kim S.-K. et al., 2023). However, memory retrieval tasks demand greater demands on attention frequency, duration, and material paradigms, while the resting state only requires being relaxed and awake, making it more convenient for data collection.

Meanwhile, D'Atri et al. (2021) revealed that EEG slowing during rapid eye movement (REM) sleep is most strongly correlated with cognitive ability, indicating that sleep metrics may serve as potential disease markers. Collecting EEG in the eyes-open resting state is more cost-effective for recognition analysis. However, inconvenient data collection during task status may be more beneficial for analysis (Li Z. et al., 2024). Therefore, careful assessment is needed to determine whether task paradigms can provide richer insights compared to resting-states, although this poses a significant challenge.

In summary, the eyes-open resting state is currently considered more convenient for data collection. However, it remains essential to further explore the subtle differences in EEG across different states in patients to enhance the understanding of cognitive states and clinical applications.

#### 4.1.2 EEG rhythms

In the rhythmic analysis for AD recognition, the focus is on single rhythms and frequency ratios. Alterations in EEG patterns within specific frequency bands seemingly reflect the progression of the disease.

Researchers have commonly found that AD participants exhibit increased δ rhythms (Zheng et al., 2023), elevated θ power, and decreased α power (Moghadami et al., 2021; Klepl et al., 2022; Simmatis et al., 2025). However, due to differences in power transformation across various populations and methodological settings, conclusions often vary. In AD groups, resting-state EEG bipolar channels show increased δ, θ, and low-α power, along with decreased high-α power (Del Percio et al., 2022; Tucci et al., 2023). High-frequency oscillations are increased in AD EEG, while low-frequency oscillations are reduced (Gaubert et al., 2019). The θ band shows significant differences in latent factor distribution between AD and the normal groups (Chamoli, 2021), facilitating AD identification. It is crucial to explore whether this finding applies to larger and more diverse samples. In the AD group, signal energy characteristics shift from high frequency to low frequency, particularly in the δ, θ, and β sub-bands (He et al., 2023). Musaeus et al. (2018) indicates that the classification performance is optimal in the θ band, underscoring its significance in AD classification. However, (Fouad and Labib 2023) argues that the β band, particularly in the central lobe, is more effective in the high-frequency range of EEG. In the AD vs. HC, β band signals have lower discriminative power than the γ band but higher than other bands (Nour et al., 2024). Conversely, Yang et al. (2024) and Acharya et al. (2025) represent α is more suitable for AD recognition. This raises a hidden question: why is there a significant discrepancy in the importance of the information captured by different rhythms in the analysis? It is likely because different rhythms capture distinct information, and EEG processing methods can also subtly affect frequency bands roles. Combining bands (δ, θ, α, and β) achieves better recognition than using a single band (Li et al., 2021), suggesting that an integrative approach may be more effective.

Power ratios facilitate comparisons of activity in specific frequency bands, enhancing the understanding of functional brain changes. Bipolar δ/α and θ/α resting EEG features demonstrate good classification performance (Lizio, 2020). Nevertheless, it is still necessary to assess whether these ratios truly encompass the neural complexity of cognitive decline. The θ-α ratio (TAR) is considered to be associated with declines in cognitive ability measured by MMSE scores (Meghdadi et al., 2024). Both AD and HC groups exhibit significant EEG slowing in the frontal TAR ratio during wakefulness and sleep, while the MCI group shows slowing only in sleep (Meghdadi et al., 2023). This ratio is not limited to contributing to AD recognition. The median θ/β ratio is significantly higher in the AD group than in HC across various durations (Miao et al., 2021). Additionally, the (α + β)/(δ + θ) power ratio is significantly reduced in AD patients (Kopčanová et al., 2024).

In summary, the rhythmic information from δ, θ, β, and the θ/α ratio may be more beneficial for EEG analysis in AD (Criscuolo et al., 2024). However, this does not diminish the significance of other bands. From a biological perspective, θ and α waves are considered rhythms closely related to cognitive function, memory, and learning within traditional frequency bands. Based on literature findings, the roles of individual θ, α, and their ratios in distinguishing AD are particularly pronounced. Among these, research on α power has gained more attention in recent years. However, this does not imply that other frequency bands lack research significance. Exploring the relationships between frequency band information across different diseases is also a promising research direction. Researchers are increasingly focusing on mid-to high-frequency bands and the integration of band information in exploring AD. Importantly, there has yet to be exploration into the impact of integrating band information on individual differences. The pursuit of consistent conclusions and robust verification must continue.

#### 4.1.3 EEG features

Rhythms and power ratios can distinguish between the EEG of AD and non-AD individuals. Undoubtedly, the various features of EEG also play a similar role. Cognitive decline leads to EEG slowing, reduced complexity, and altered in brain connectivity compared to HC (Chamoli, 2021; Ding et al., 2022).

In the temporal analysis of EEG, wavelet coherence, fractal dimension, quadratic entropy, wavelet energy, quantile plots, and visibility graphs are actively applied (Vicchietti et al., 2023). Among these, wavelet coherence and quantile plots more reliably distinguish between AD and healthy elderly subjects. However, it's worth considering whether relying on a particular method might overlook other potentially informative metrics. Regarding skewness, kurtosis, and Hjorth features (activity, mobility, complexity), Hjorth features capture information more effectively. At this time, another type of temporal information is thought to play a more crucial role. The study revealed a significant decrease in Hjorth activity on the α and β in AD compared to controls, while Hjorth activity in the γ band was increased considerably (Nedeljković, 2023).

In terms of nonlinear parameters for quantifying EEG complexity and irregularity, AD patients exhibit lower values of LZ complexity (Ding et al., 2022), SampEn, FuzzyEn, PE, and multiscale permutation entropy (MPEs) compared to HC, while their self-mutual information values are higher (Ruiz-Gómez et al., 2018a). However, Gaubert et al. (2019) indicates that EEG complexity may be enhanced in AD. This suggests that researchers' understanding of complexity may be more nuanced than previously thought. During the MCI stage, there is a mix of both high and low PE values (Şeker et al., 2021). There are also significant differences between MCI and normal controls (NC) (Puri et al., 2023). Ruiz-Gómez et al. (2018b) suggests that cross-sample entropy (cross-SampEn) effectively characterizes neural coupling patterns in MCI and AD. Nobukawa et al. (2020) confirmed that recognizing complexity (multiscale entropy and SampEn) is accurate in distinguishing AD from HC, while functional connectivity in the α, β, and γ bands (measured with PLI) shows even higher accuracy. Brain network connectivity properties are closely associated with neurological disorders, indicating the strong potential of spatial information for multi-information classification (Goerttler et al., 2024). In resting-state EEG, AD patients show reduced synchrony (Zheng et al., 2023). Functional connectivity of nearly all channel pairs is significantly lower in the AD group compared to non-AD, with greater differences observed in α than in δ band (Yu et al., 2024). Additionally, the coherence in β is increased (Jiang et al., 2025). However, the implications of these changes require further study.

In dementia research, AD exploration has predominated, though other types like DLB, FTD, and MCI are also considered. The overall similarity between brain regions in MCI and AD decreases, particularly in β1, with abnormal coupling in low-frequency bands (θ and α) (Ruiz-Gómez et al., 2018b). Dominant rhythms (DR) slow down in both AD and DLB, with DLB showing a more pronounced slowdown. Notably, AD is characterized by increased δ coherence, decreased α power, and reduced prominence of DR frequency (Meghdadi et al., 2021a). Additionally, AD shows stronger δ-α and δ-β connectivity compared to FTD (Si et al., 2023). Existing research indicates that there are indeed close connections between different types of dementia. To break these connections, the key to identification lies in extracting spatial domain information from feature information in specific frequency bands. However, the calculation of connectivity often depends on the number of channels. Limited channel information may lead to a reduction in EEG connectivity data. This highlights the limitations of the research, underscoring the urgent need for an accurate consideration of multi-layer features to facilitate differentiation between diseases.

In summary, different groups exhibit distinct characteristics, with complexity, nonlinear parameters, and connectivity providing richer features for classification. Findings in δ and α bands are more frequently discussed, influencing the capture of relevant target information. Integrating multiple features can further enhance recognition for optimal outcomes. Despite extensive research on bands and features, widely accepted conclusions remain lacking. Future studies should prioritize a deeper exploration of EEG biomarkers of disease progression, integrating multiple features while considering the individual variability and methodological differences that could impact results.

### 4.2 Brain regions

There are notable differences in the connectivity of the entorhinal cortex and parahippocampal region in individuals with AD (Ranjan and Kumar, 2023). The EEG from different brain regions contributes variably to AD detection. (Gaubert et al. 2019) indicates that neurodegenerative processes significant impact EEG indicators in the frontocentral region. All brain rhythms in the frontal lobe exhibit high sensitivity to AD. Faster rhythms (α and β) affect the parietal lobe, while slower rhythms (δ) spread along the temporal lobe to the occipital region (Song et al., 2022). It highlights the importance of studying rhythms in different brain regions. Coherence among EEG channels in the frontal-parietal region increases (Tzimourta et al., 2019; Gunawardena et al., 2023). Nonetheless, an increase in coherence does not necessarily indicate an improvement in cognitive function; rather, it may reflect compensatory mechanisms in neurodegenerative diseases. EEG channels in the parietal and occipital regions significantly contribute to functional connectivity, with increased coherence observed in the parietal-occipital region of AD patients. Overall, AD cases display increased connectivity, while HC exhibit sparser connectivity with fewer densely connected areas (Moghadami et al., 2021). These differences raise questions among researchers about the mechanisms driving these variations and whether current EEG can effectively capture the complexity of AD. Both groups show a distinct cluster of dense connectivity nodes in the central parietal and occipital regions, along with some strong edge effects in the frontal and temporal areas (Klepl et al., 2023). The parietal, temporal, and frontal lobes are the most affected regions in all EEG assessments (Rodrigues et al., 2021; Jain and Srivastava, 2025). Laton et al. (2022) found that AD impacts the temporal lobe in the early stages, subsequently affecting the parietal and frontal lobes as the disease progresses. This progressive research emphasizes the need for longitudinal studies, as the limitations of cross-sectional studies become increasingly apparent.

The relative power of β2 in the frontal lobe for HC and the β1 power in the right temporoparietal region for aMCI were identified by Farina et al. (2020) as the best predictive indicators. Nevertheless, these indicators have not yet been validated in clinical settings, especially for different populations. Clinical corrections indicate that AD is associated with the central brain, temporal lobe, and parietal regions, aligning with previous research findings (Gore and Rathi, 2022). In medical contexts, the most significant impact of AD-related neurodegeneration (considering cerebrospinal fluid, EEG, and APOE indicators) on EEG metrics is found in the parietooccipital region (Jiao et al., 2023). Frequency analysis reveals phenomena such as increased θ waves in the frontal/temporal lobes and decreased β waves in the central parietal region (Kim N. H. et al., 2023). However, another finding indicates that in AD and FTD patients, frontal θ connectivity is reduced, while parietal and occipital β connectivity is enhanced (Zheng et al., 2024a). This contradiction may arise from differences in research methods, significant variability in sample sizes, or other unobserved physiological mechanisms. The specific connections of frequency band information in brain regions of AD still require further exploration.

The temporal and occipital-parietal regions significantly contribute to distinguishing between HC and severe AD, particularly through channels T5, T6, O1, and O2 (Fan et al., 2018). Al-Nuaimi et al. (2021) notes that channels in the temporal and parietal regions (T6, T3, T5, and P3) are more suitable for AD identification. Similarly, T5 and P3 are recognized as key distinguishing channels for AD (Jain and Srivastava, 2025). In recent research, Siuly S demonstrated that β and γ rhythms in channels Cz, F4, P4, T6, and Pz are the most reliable biomarkers for recognizing AD (Siuly et al., 2024). The Cz and Pz channels exhibit significant differences in AD EEGs and can be considered fundamental regions where most changes occur. However, Taub and Savir (2024) suggests that O1 and O2 are more important in HC, while C3 and C4 are more significant in AD EEG. Currently, there is no consensus on these findings (Rostamikia et al., 2024), indicating a critical need for standardized methodologies in EEG analysis.

In summary, based on the endogenous pathological changes associated with the disease, the following acceptable phenomena can be observed in several brain regions. Compared to HC, all EEG rhythms in the frontal lobe of patients with cognitive decline show significant changes, accompanied by pronounced slow waves. The temporal lobe has been reported as the first affected region, with research on this area increasingly focusing on the power ratios between rhythms. Although there is controversy regarding the changes in individual rhythms, it is generally accepted that a reduction in power is the predominant phenomenon. In patients with MCI and AD, the Hjorth mobility in the parietal and occipital lobes decreases due to a reduction in neuronal oscillation frequencies linked to cognitive decline. The θ (absolute/relative PSD) spectral values in the occipital region exhibit a monotonic trend from HC to MCI and AD. In conclusion, cognitive decline not only leads to reduced functional connectivity in the brain but also causes fluctuations in power, reflecting internal self-damage within the brain.

In brief, neurodegenerative changes in AD can locally impact EEG activity, evolving into more complex functional impairments. Understanding these changes is crucial for elucidating EEG neuropathological mechanisms of AD. However, studies often have inconsistent interpretations of contributions from different brain regions. The frontal, parietal, temporal, and parieto-occipital lobes are effective areas for AD analysis. Exploring EEG in these regions enhances our understanding of AD-related lesions. Strengthening the comparison of the contribution weights between brain regions in distinguishing AD types will be more meaningful.

### 4.3 Model analysis

Patient compliance issues have hindered data collection, resulting in small sample sizes. Data augmentation techniques can address the issue. Researchers often utilize overlapping and non-overlapping sliding windows to increase the amount of data (Tzimourta et al., 2019; Kayasandik et al., 2022; Xia et al., 2023; Alessandrini et al., 2022; Jain and Srivastava, 2025). However, this method has limitations with very small samples. However, this method has limitations, especially when the sample size is very small, as the variability in the data cannot be adequately captured. This raises concerns about the generalizability of results from small samples, leading to a gradual shift toward in-depth research on data augmentation methods. Song et al. (2021) employed Variational Autoencoders (VAE) and Generative Adversarial Networks (GAN) for data augmentation, demonstrating the validity of generated data in brain network feature space. Subsequently, they applied GAN-based adaptive learning to mitigate the limited samples in deep learning while also overcoming the sparsity of high-level features, successfully classifying of AD (Song et al., 2022). It is important to note that this technique can easily lead to overfitting, particularly if the generated data does not accurately reflect the meanings of samples in real-world environments. Research on actual samples from big data will be more meaningful. As dataset grows, dimensionality reduction methods like PCA become necessary to reduce complexity, and PCA has gained popularity among researchers (Fouad and Labib, 2023; Calub et al., 2023; Biagetti et al., 2021; Tang et al., 2022; He et al., 2023). The application of dimensionality reduction methods requires caution, as discarded information may affect the performance of subsequent models. If possible, combining feature contribution weights to reasonably retain valuable information while achieving dimensionality reduction can better ensure data reliability.

Additionally, analysis is not confined to directly modeling EEG features or raw data. After feature extraction, a preliminary selection of relevant features can occur (Taub and Savir, 2024). However, raw data can be processed through deep models before being input into classifiers for identification. This dual approach emphasizes the importance of flexibility in data processing, allowing both high-dimensional raw data and the refined feature set to be effectively utilized. In summary, prior to data analysis, models can be employed for data reduction, augmentation, and selection, followed by recognition and analysis.

AD identification depends on classifiers and statistical analysis methods. Traditional machine learning techniques, like SVM, ANN, and KNN, are frequently utilized in AD studies and demonstrate recognition capabilities comparable to deep learning models (Kayasandik et al., 2022; Gopu et al., 2024). With the advancement in deep learning, variations of CNN (Bi and Wang, 2019) and LSTM networks (Gkenios et al., 2022; Siuly et al., 2024) have proven effective for AD recognition. However, the complexity of the model may also lead to overfitting, so independent datasets are needed for careful validation. Additionally, designing feature extraction network models can also aid in the distinctive analysis of EEG information related to AD (Amato et al., 2024; Dogan et al., 2023; Zhao et al., 2019). Furthermore, a range of innovative models continues to emerge. Researchers are currently focused on developing new models that can capture more information while ensuring robustness and accuracy in identification (Yu et al., 2019; Ge et al., 2020; Tang et al., 2022; Chen et al., 2023; Bhendare and Dewangan, 2023; Mohammed, 2024).

An analysis of the recognition performance presented in Table 3 reveals that articles demonstrating high recognition accuracy predominantly utilize innovative fusion models. Certainly, the choice of model depends on the form of data analysis. The combination of these models with modern techniques may provide valuable insights. Future developments should not overlook traditional models. Clearly identifying important EEG information during recognition will enhance the value of EEG identification for AD.

These conclusions are based solely on the authors' research methods. The ambiguities raise questions about their generalizability, reflecting the current confusion in AD identification. Dependence on datasets and methods may limit the generalizability of research findings. Consequently, research on the diversity of EEG biomarkers for AD would benefit from clear guidance, improving the consistency of findings and their clinical applicability. Establishing standardized protocols for data collection, augmentation, and analysis can significantly enhance the robustness of future research.

The primary objective of this research is to translate findings into clinical practice.

Current studies have achieved high recognition accuracy across various traditional and innovative models, particularly in categories two, three, and four, where the classification accuracy for minority classes has reached 80%, no longer presenting a challenge. However, these high-performance models are heavily reliant on preliminary data processing, including paradigm design, data collection, and analytical methods, all of which significantly impact the final recognition performance. During the implementation of paradigms and data collection, the influence of subjective factors cannot be overlooked. Research must consider not only the need for a reasonable and suitable environment but also the individual characteristics of the subjects. This is especially true in clinical settings, where individual differences become more pronounced, and data collection may face greater challenges. Therefore, there is an urgent need to explore and develop AD recognition models that can operate across different datasets to facilitate the widespread application of clinical models. Although current AD recognition models demonstrate high accuracy within the datasets discussed, their performance may decline when transferred to other datasets. This highlights the importance of enhancing model robustness to ensure broad applicability. Furthermore, a critical aspect of model deployment in clinical practice is robustness testing, as patients' disease manifestations often accompany anomalies, and the quality of clinical data can be affected by various unpredictable factors.

In summary, if these models are to be promoted for clinical application, many limitations still exist. There is an urgent need to strengthen model validation through generalizability and robustness testing across datasets. Future research should focus on improving the consistency of data collection and exploring more representative datasets to further enhance the clinical applicability and reliability of the models.

### 4.4 Other types of biomarkers

In addition to amyloid proteins, which can serve as direct biomarkers for AD in a medical context, other forms of neurological manifestations will also aid in the exploration of AD biomarkers.

**Event-related potentials (ERP):** ERP is distinct from EEG as a specific type of brain-evoked potential. Abnormal total δ and θ event-related oscillations may aid in the early detection of MCI, while δ responses can indicate the transition to AD (Tülay et al., 2020; Güntekin et al., 2022). One conclusion suggests β PSD in the prefrontal cortex is a potential marker for AD risk (Li Z. et al., 2024).

**Transcranial Magnetic Stimulation (TMS):** TMS-EEG can replace traditional neuroimaging methods in the diagnosis of AD, providing clinicians with pathophysiological features related to functional and neuronal communication changes (Tăuţan et al., 2022b; Tăuţan et al., 2023). The maximum amplitude after TMS, Hjorth complexity, and the amplitude of the TEP following stimulation are the most relevant features for distinguishing AD from HC. The time taken for post-stimulation EEG to return to baseline can also reveal important information about the pathology (Tăuţan et al., 2022a). Although authors achieved higher accuracy in ROIs on the right central, right parietal, and right frontal lobes using this biomarker, a definitive conclusion regarding whether the right or left hemisphere is more dominant in AD has yet to be reached (Tăuţan et al., 2023).

**Magnetic Resonance Imaging (MRI):** In the classification of AD using resting-state EEG (rsEEG), resting MRI (sMRI), and rsEEG + sMRI, multiple studies have concluded that individual MRI features outperform EEG features, with both exhibiting moderate recognition capabilities (Jesus et al., 2021; Farina et al., 2020; Waser et al., 2019; Ferri et al., 2021). Moreover, the combined markers from both modalities can capture multidimensional information, leading to higher identification rates (Li J. et al., 2024; Liu et al., 2025). However, in the case of studies tracking cognitive decline, the accuracy of EEG recognition related to neurophysiological oscillation mechanisms that support cortical arousal and quiet vigilance regulation matches the accuracy achieved by enriched CSF and sMRI biomarkers (Babiloni et al., 2024).

**Functional Near-Infrared Spectroscopy (fNIRS):** Similar to MRI, a combined EEG-fNIRS feature set achieves higher accuracy by leveraging their complementary characteristics compared to using EEG or fNIRS alone (Ho et al., 2021b). Currently, fNIRS provides phenomenological representations is a mainstream trend (Xie et al., 2024).

EEG's convenience and low cost make it popular among researchers. However, combining various information can enhance AD recognition and enable earlier disease screening, promoting better health development.

Through the discussions above, key findings have been presented in recent years. This review aims to provide foundational insights for more researchers interested in EEG-based AD analysis, encouraging further exploration of specific areas of interest. From the typical prospects presented in Table 3, it is evident that future research on AD discrimination can be conducted from multiple angles:

**Data Perspective:** An in-depth exploration of the coupling of information across EEG signal frequency bands is expected to reveal potential biomarkers. Integrating features can capture more valuable information and enhance the effectiveness of discrimination. Expanding datasets and increasing the sample size of longitudinal studies can reduce individual variability and improve tracking and predictive capabilities. Utilizing multimodal data can provide additional advantageous information from various brain sources. **Model Perspective:** Ensemble models can be constructed to either expand the model's scope or reduce label dependencies, focusing on validating the model's robustness using clinical data. **Furthermore**, designing subjective paradigms to assess cognitive abilities could pave new avenues for the development of novel therapeutic strategies. It cannot be ignored that individual brain neurodevelopment and pathology are indeed influenced by certain demographic variables and other external factors. To better evaluate the data, demographic information of different categories of subjects needs to be balanced to eliminate potential bias.

## 5 Conclusion

### 5.1 Overview of the review

**Figure 14:**
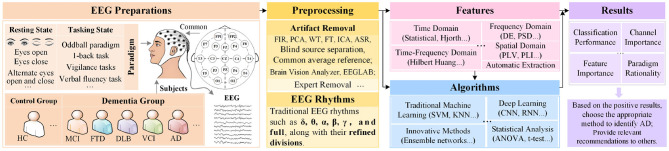
Schematic diagram of the EEG analysis process for dementia.

This study explores the application of EEG analysis in identifying AD. It provides an overview of the overall process for implementing AD recognition through EEG processing. The focus is on summarizing the current state and trends of research regarding the types of subjects addressed in the literature, the design of EEG collection experiments, preprocessing, rhythm segmentation, feature extraction, and EEG recognition methods, among others. Each stage plays a crucial role in EEG-based AD identification. Figure 14 briefly shows a schematic diagram of the EEG analysis process for dementia. From the perspective of EEG analysis, this paper discusses the significant conclusions from each section and offers suggestions for future research.

### 5.2 Trends

Based on the above, the development trends in EEG-based identification of AD can be summarized in the following aspects:

1. Neural detection technology for AD is developing in being non-invasive and low-cost.
2. Research on EEG detection for AD is delving deeper into various rhythms, features, and brain regions to identify EEG biomarkers strongly correlated with Alzheimer's. The goal is to enhance recognition performance and effectiveness, providing a solid foundation for the diagnosis and intervention of the disease. In particular, the multimodal feature analysis and recognition methods will receive significant attention. In addition, the design of the acquisition paradigm and the documentation of the subject's background need to be considered to assess their potential impact on the research results.
3. Single-modal AD recognition plays a crucial role in diagnosis and intervention. Although the combined utilization – of multiple modalities (EEG, TMS, MRI, fNIRS) for AD recognition has shown effectiveness, it remains an area of ongoing exploration. In the future, the effective integration of modalities may further advance the development of AD detection technologies.
4. In the medical field, there is an urgent need to explore EEG-based personalized and real-time monitoring models for AD recognition. The validation of clinical models necessarily involves testing for generalizability and robustness; therefore, exploring cross-dataset models will be a significant direction.

EEG-based identification of AD holds great potential for clinical auxiliary diagnosis, but it also presents significant challenges. In the research field, a deep analysis of crucial brain physiological information closely related to AD can provide important insights for more effective models of AD recognition. Clinically, personalized automated diagnostic models will help promote the application of EEG-based AD identification.

## Data Availability

The original contributions presented in the study are included in the article/Supplementary material, further inquiries can be directed to the corresponding authors.
