# Supplementary material for: The EEG analysis and identification of Alzheimer's disease: a review
Source: Front Aging Neurosci. 2025 Dec 9;17:1686628. doi: 10.3389/fnagi.2025.1686628 (PMC12722947; doi:10.3389/fnagi.2025.1686628)
Supplement: Supplementary file 1 [file Table_1.pdf]

Supplementary Material

SUPPLEMENTARY MATERIAL

Supplementary materials in Section 3.4.2 EEG Feature.

Table S1. The features involved consist of the following main characteristics based on four domains in LC-I

Domains	Features
Time	The primary focus is on statistical feature extraction. However, there are also methods that capture nonlinear features from time series data and analyze microstate information.<br><br>LC-I mainly includes: maximum value, minimum value, mean value, standard deviation, skew and kurtosis, variance, energy mean, interquartile range (IQR), EEG amplitude change (△EEGA), zero crossing interval (ZCI), Higuchi fractal dimension (HFD), Katz fractal dimension, Lempel-Ziv complexity (LZC), Hurst exponent HE, box dimension (BD), Hjorth parameter (Hjorth activity, mobility and complexity), Tsallis entropy (TsEn), approximate entropy (ApEn), sample entropy (SampEn), fuzzy entropy (FuzzyEn), permutation entropy (PE), Shannon entropy (SE), multiscale sample entropy (MSEnt), Renyi PE (RPE), multiscale wavelet entropy, cross approximate entropy, cross sample entropy, quadratic entropy, complexity value EpEn, microstate measures (lifetime, occurrence rate, converting rate), microstate analysis (global field power GFP, minimized global map difference GMD, LZ Complexity measurement signal), time-dependent power spectrum descriptor (TD-PSD), modulation frequency "patch" characteristics, spectral power Modulation Frequency "Patches", etc.
Frequency	The emphasis of the analysis is on power and spectral characteristics, along with an examination of energy-related features.<br><br>LC-I mainly includes: Power Spectral Density (PSD), average power spectrum, absolute power, relative power (RP), absolute power, spectral power, relative power, spectral entropy (SE), diffusion entropy, differential entropy (DE), median frequency (MF), amplitude square coherence of power spectrum, event-related spectral perturbation (ERSP) characteristics, spectral measurement analysis, dominant rhythm (DR), DR frequency domain prominence, spectral coherence, median spectral frequency (MSF), SPR spectral power ratio, Fourier transform, spectral power density (calculated by fast Fourier transform, such as FFT, Welch algorithm, Hanning window, no phase shift, etc.), potential energy (band energy) extracted by Fourier transform, information energy, etc.
Time-Frequency	Wavelet transform is dominant, and its derived features are also actively analyzed.<br><br>LC-I mainly includes: discrete wavelet transform, continuous wavelet transform, generated adaptive wavelet, wavelet coherence, small wavelet energy, self-mutual information (AMI), rational asymmetry (RASM), differential asymmetry (DASM), amplitude modulation rate of change (Hilbert Huang calculation), Hilbert transform (HT), total activity, phase-locked activity, and non-phase-locked activity, lacstral analysis (the square amplitude of the inverse of the DWT coefficient logarithm of the signal), and algorithm complexity, etc.
Spatial	This field focuses on network analysis, encompassing functional connectivity, effective connectivity, and graph analysis. Additionally, analyses of coherence are also of interest.<br><br>LC-I mainly includes: resting state network (RSN), functional connectivity (correlation coefficient, mutual power spectral density), coherence analysis, source analysis of eLORETA, phase synchronization index (PSI), visibility graphs (network related), phase lag index (PLI) (functional connectivity), Pearson correlation, Spearman correlation, minimum redundancy maximum correlation, phase lag index (PLI), weighted phase lag index (wPLI), phase locking value (PLV), mutual information (MI), amplitude envelope correlation (AEC), spectral coherence, imaginary part of coherence, some graph theory features (clustering coefficient, characteristic path length, local efficiency, global efficiency, parameter coefficient Pc), etc.

It is important to note that the features mentioned above have been widely analyzed in literature within the scope of this study; however, this does not imply that the EEG characteristics for AD analysis are limited to these. The above features are provided for researchers' reference only.
